# Global-scale variability of glacier velocity and mass loss across terminus environments

**DOI:** 10.1038/s41467-026-78654-y

**Published:** 2026-10-23

**Authors:** Joseph Mallalieu, Jonathan L. Carrivick, Matthias Huss, Iestyn D. Barr, Nicholas E. Barrand, Cassandra L. Raby

**Affiliations:** 1 School of Geography, Earth and Environmental Sciences, University of Birmingham, Birmingham, UK; 2 School of Geography and water@leeds, University of Leeds, Leeds, UK; 3 School of Biology, University of Leeds, Leeds, UK; 4 Laboratory of Hydraulics, Hydrology and Glaciology (VAW), ETH Zurich, Zurich, Switzerland; 5 Swiss Federal Institute for Forest, Snow and Landscape Research (WSL), Sion, Switzerland; 6 Department of Natural Sciences, Manchester Metropolitan University, Manchester, UK

**Keywords:** Cryospheric science, Climate and Earth system modelling, Geomorphology

## Abstract

The environment in which a glacier terminates can strongly influence its behaviour, stability and future evolution, yet the relationship between terminus type and glacier dynamics at the global scale remains poorly constrained. Here we present a globally-complete classification of glacier termini distinguishing between land, lake, marine and shelf environments, and integrate it with datasets of glacier geometry, dynamics and climate. We show that although lake-, marine- and shelf-terminating glaciers comprise only 3.2% of glaciers globally, they account for over half of global glacier area and mass loss. We also show that lake- and marine-terminating glaciers flow, on average, 10% and 44% faster than land-terminating glaciers, respectively. Furthermore, specific (area-normalised) mass loss rates at lake-terminating glaciers are 57% greater than those at land- and marine-terminating glaciers. Our findings underscore the need for improved representation of non-land glacier termini in models of glacier evolution and sea-level rise, and suggest that distinguishing the environmental and geometric controls of termini on glacier dynamics remains an important challenge for future research.

## Abstract (web summary)

Despite their low abundance, glaciers terminating in lakes, oceans or ice shelves account for over half of global glacier area and mass loss, and lake-terminating glaciers lose mass at rates 57% greater than land- and marine-terminating glaciers.

## Introduction

Constraining environmental controls on glacier dynamics and mass balance is critical for accurately projecting future glacier response to climate change and associated impacts on downstream hydrology, ecosystems and sea-level rise^1–4^, particularly in light of globally-accelerating glacier mass loss^5–7^. On decadal to centennial timescales, glacier evolution and dynamics are largely determined by climate, basal topography and substrate, and their respective feedbacks with glacier properties including ice thickness, thermal regime and hydrology^8^. Glaciers terminating in marine or lake settings are subject to additional thermo-mechanical forcings, including iceberg calving, subaqueous melt, and variations in subglacial hydrology and basal shear stress^9–11^. These processes modify terminus geometry and, through their influence on longitudinal driving stresses^12^, can affect glacier dynamics and surface-elevation extensively upstream of the terminus^13–17^. Thus, the environment in which a glacier terminates is an important control on its dynamics, stability and future evolution.

Mountain glacier evolution models have, until recently, typically relied on climate model forcings and representations of surface mass balance processes, omitting the role of ice-dynamics^18,19^. Now, the growing availability of global glacier datasets, including geometry^20,21^, ice thickness^22^, velocity^23^, and mass change^5^, together with increased computational power, is enabling flowline approaches that incorporate representations of ice dynamics^19,24,25^. Regional observations indicate that lake- and marine-terminating glaciers can exhibit distinct dynamic behaviours relative to their land-terminating counterparts, including faster ice flow and enhanced dynamic thinning^17,26–31^, but the global-scale expression of these differences remains unquantified. Furthermore, many regional and global-scale models of glacier evolution neglect lake-terminating glacier dynamics and oversimplify those of marine-terminating glaciers^19,32^, while existing classifications of glacier terminus types worldwide are incomplete and inconsistent. A globally complete classification of glacier termini and an assessment of their relationship with glacier dynamics are therefore urgently needed, particularly as glaciers increasingly transition between terminus types in response to atmospheric and oceanic warming^33–36^.

Here we present a globally complete classification of glacier termini that distinguishes between land, lake, marine and shelf environments. We integrate this classification with key datasets of glacier properties and climate published for the ~216,000 glaciers in the Randolph Glacier Inventory (RGI) 6.0^20^ to quantify the associations between terminus type and glacier dynamics at the global scale. We find that although the vast majority of global glaciers are land-terminating, lake-, marine-, and shelf-terminating glaciers collectively account for over half of global glacier area and mass loss. We also show that terminus types are associated with different velocity regimes and specific mass change rates, and that these patterns persist after controlling for other key physical and climatic drivers of glacier dynamics. Our results highlight the need for intensified monitoring and representation of non-land glacier termini in models of glacier evolution and sea-level rise.

## Results

### Classification and distribution of glacier termini

**Fig. 1:**
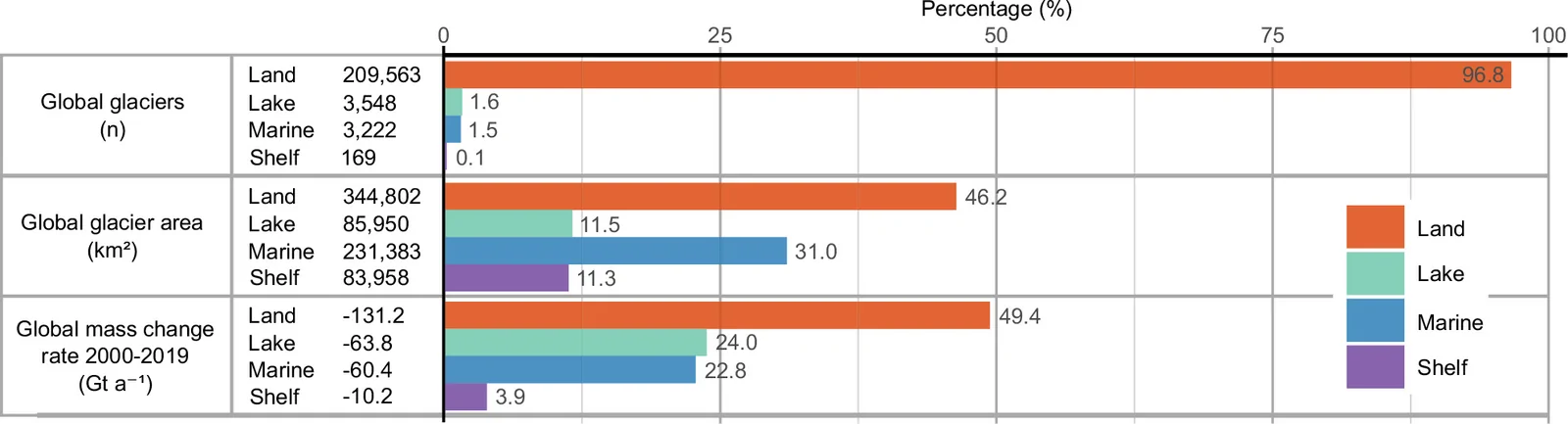
Global classification of glacier termini by type. Absolute and percentage values of glacier number, area, and mass change rate (2000–2019) attributed to land-, lake-, marine-, and shelf-termini for all RGI 6.0 glaciers. Glacier numbers and areas from RGI Consortium^20^, mass change data from Hugonnet et al.^5^.

Globally, 96.8% of all RGI 6.0 glaciers terminate on land. Lake- and marine-termini represent 1.6% and 1.5% of glaciers, respectively, while shelf-termini account for 0.1% (Fig. 1). However, over half of global glacier area drains to non-land termini, specifically 31.0% to marine-, 11.5% to lake-, and 11.3% to shelf-termini. Almost half (49.4%) of global glacier mass loss (2000-2019 mass change rate, from ref. ^5^) occurred at land-terminating glaciers, with 24.0% and 22.8% at lake- and marine-terminating glaciers respectively, and 3.9% at shelf-terminating glaciers (Fig. 1).

Regionally, land-termini predominate, but lake-termini occur in all RGI regions, with relatively high concentrations (~7–10%) in the Russian Arctic, Scandinavia, and Iceland (Supplementary Table 1). Lake-termini are most abundant in the Greenland Periphery (*n* = 754), with more than 300 also present in the Western Canada and USA, Arctic Canada South, Central Asia, South Asia East, and the Southern Andes regions. Marine-termini are typically confined to higher latitudes (occurring in 9 of 19 regions), and account for 48.7% of termini in the Antarctic and Subantarctic, and 35.1% in the Russian Arctic, with the greatest abundance in the Antarctic and Subantarctic (*n* = 1339) and Greenland Periphery (*n* = 903). Shelf-termini only occur in the Russian Arctic, and the Antarctic and Subantarctic (Supplementary Table 1).

**Fig. 2:**
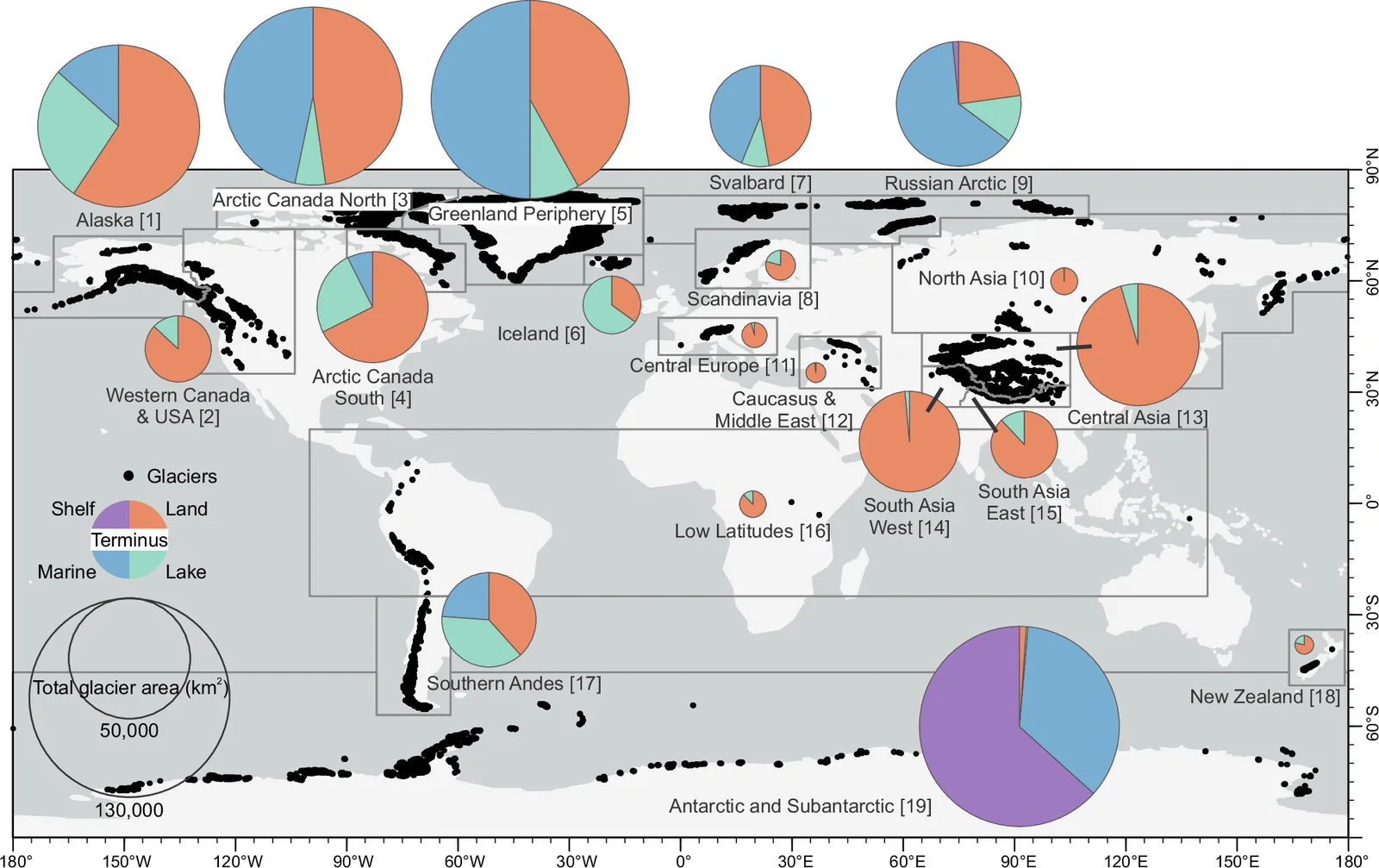
Distribution of glacier area by terminus type across RGI 6.0 world regions. Discs illustrate the proportion of glacier area draining to land-, lake-, marine- and shelf-termini. Disc area is scaled to represent the total glacier area in each corresponding region. Glacier locations are shown in black. Values in square brackets correspond to RGI region numbers. Glacier areas from the RGI Consortium^20^.

In lower latitudes, glacier area predominantly drains to land-termini (exceeding 87% in each of RGI regions 11-16), whereas at higher latitudes an increased proportion drains to other terminus types (Fig. 2 and Supplementary Table 2). Marine-termini drain 63.2% of glacier area in the Russian Arctic, ~45% in the Arctic Canada North, Greenland Periphery, and Svalbard regions, and 36.2% in the Antarctic and Subantarctic. Lake-termini drain 65.1% of glacier area in Iceland, 37.8% in the Southern Andes, and more than 21% in the Alaska, Arctic Canada South, Scandinavia, and New Zealand regions. Shelf-termini drain 1.6% and 62.6% of glacier area in the Russian Arctic, and the Antarctic and Subantarctic, respectively (Supplementary Table 2). Regional patterns in the 2000–2019 mass change rate^5^ broadly replicate those of glacier area, with land-termini typically dominating regional mass loss in the lower latitudes (Supplementary Table 3). In contrast, non-land termini dominated regional mass loss in Iceland, the Southern Andes, Svalbard, and the Russian Arctic, where over 50% of losses occurred at lake- or marine-terminating glaciers. Overall, lake-terminating glaciers accounted for more than 20% of glacier mass loss in 7 of 19 regions, and marine-terminating glaciers for more than 30% in 5 of 19 regions (Supplementary Table 3).

### Glacier dynamics across terminus types

**Fig. 3:**
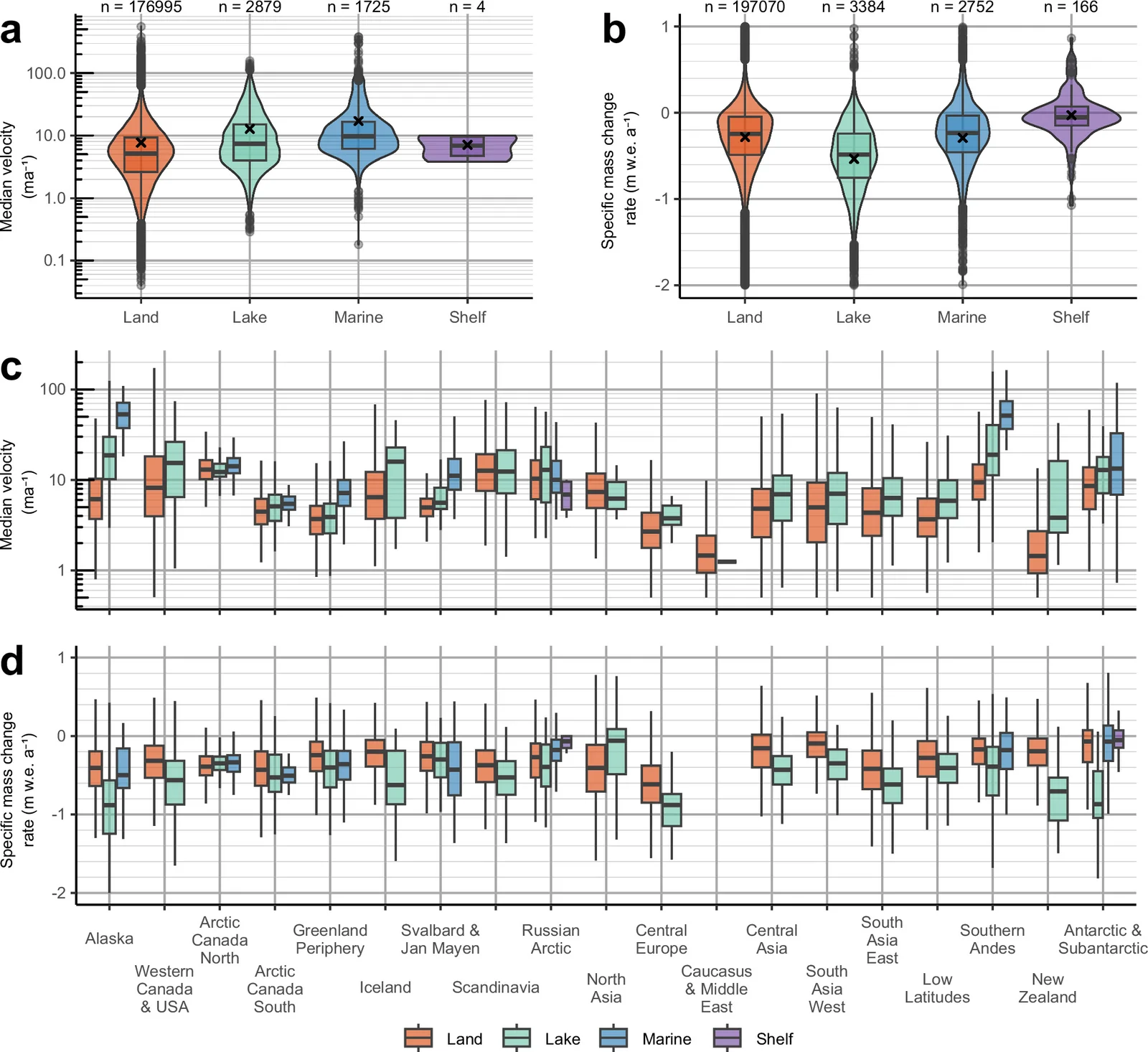
Global and regional glacier dynamics by terminus type. Global distributions of median glacier velocity (**a**) and specific mass change rate (2000-2019) (**b**), and corresponding distributions across RGI 6.0 world regions (**c, d**). Violin plots and black crosses in (**a**, **b**) show data distributions and mean values, respectively. Box plots in (**a–d**) show median values and interquartile ranges. To improve clarity, the y-axis in (**b**) is cropped, omitting 1,189 (0.6%) outlying values. Outliers are also not plotted in (**c**, **d**). The Caucasus and Middle East region is omitted from (**d**) due to the absence of specific mass change rate data corresponding to RGI 6.0 glacier outlines. Velocity data adapted from Millan et al.^23^; mass change data from Hugonnet et al.^5^.

Glacier properties exhibit a continuum across land-, lake-, marine- and shelf-termini - typically increasing in size (area, length and thickness) and latitude, and decreasing in median elevation and slope - with lake-termini occupying intermediate values between land- and marine-termini (Table 1 and Supplementary Fig. 1a–h). Climatically, land-, lake- and marine-termini are relatively indistinct, but shelf-termini are located in colder, drier climates (Table 1 and Supplementary Fig. 1i, j). Velocity and specific mass change rate data were available for 83.9% (*n* = 181,603) and 93.9% (*n* = 203,372) of the 216,502 glaciers in RGI 6.0, respectively. Globally, median glacier velocities (2017-2018) were lowest at land-terminating glaciers (5.1 m a^−1^), intermediate at shelf- and lake-terminating glaciers (7.2 and 7.4 m a^−1^ respectively), and highest at marine-terminating glaciers (9.7 m a^−1^) (Table 1 and Fig. 3a). Regionally, median velocities at marine-terminating glaciers typically exceeded those of lake-terminating glaciers, which in turn outpaced land-terminating glaciers (Fig. 3c). Globally, median specific (i.e. area-normalised) mass change rates (2000–2019) were negative across all terminus types. The greatest mass losses occurred at lake-terminating glaciers, with a median specific mass change rate of −0.490 m w.e. a^−1^, roughly double that of land- and marine-terminating glaciers (−0.245 and −0.239 m w.e. a^−1^ respectively), while shelf-terminating glaciers exhibited the lowest median mass losses (−0.051 m w.e. a^−1^) (Table 1 and Fig. 3b). This pattern persists regionally, with lake-terminating glaciers exhibiting more negative median specific mass change rates than land- or marine-terminating glaciers in 15 of the 18 regions with available data (Fig. 3d).

**Table 1:** Median values of key glacier properties by terminus type for RGI 6.0 glaciers

Glacier property	Terminus type				All glaciers
	Land	Lake	Marine	Shelf	
Absolute latitude (°)	43.5 (43.4–43.5)	59.9 (59.0–60.1)	67.8 (67.5–68.1)	71.9 (71.6–72.2)	43.8 (43.8–43.9)
Polar deviation (°)	52 (51–52)	46 (44–48)	91 (87–96)	97 (84–109)	52 (52–52)
Median elevation (m a.s.l.)	3389 (3344–3433)	1352 (1305–1398)	415 (375–426)	275 (225–308)	3182 (3144–3224)
Elevation range (m)	321 (319–322)	546 (527–564)	614 (574–660)	430 (300–520)	324 (323–326)
Area (km^2^)	0.24 (0.24–0.24)	2.00 (1.84–2.20)	6.43 (5.95–7.20)	197.77 (109.61–266.98)	0.25 (0.25–0.25)
Maximum surface flowline length (m)	704 (700–707)	2257 (2116–2402)	3600 (3349–3812)	10924 (8068–13208)	718 (714–722)
Debris cover (%)	10.4 (10.3–10.5)	8.1 (7.6–8.5)	0.9 (0.8–1.0)	0.3 (0.1–0.8)	10.1 (10.0–10.2)
Slope (°)	24.0 (24.0–24.0)	16.4 (16.1–16.7)	12.5 (12.1–12.8)	2.8 (2.3–3.6)	23.8 (23.8–23.9)
Median thickness (m)	30.7 (30.6–30.8)	60.8 (58.5–62.7)	90.0 (86.1–93.5)	208	31.2 (31.0–31.3)
Median velocity (m a^−1^)	5.1 (5.1–5.2)	7.4 (6.9–7.7)	9.7 (9.2–10.1)	7.2	5.2 (5.2–5.2)
Specific mass change rate 2000–2019 (m w.e. a^−1^)	−0.245 (−0.247–−0.243)	−0.490 (−0.508–−0.479)	−0.239 (−0.255–−0.222)	−0.051 (−0.080–−0.012)	−0.248 (−0.250–−0.247)
Mean annual air temp. 1991–2020 (°C)	−6.2 (−6.2–−6.1)	−5.5 (−5.8–−5.2)	−7.0 (−7.3–−6.5)	−13.4 (−14.1–−12.8)	−6.2 (−6.2–−6.1)
Total annual precip. 1991–2020 (mm)	954 (948–958)	1122 (1073–1160)	890 (823–956)	548 (477–642)	955 (950–958)

Values in brackets indicate 95% bootstrap confidence intervals (CIs). CIs for velocity and thickness are omitted for shelf-terminating glaciers due to limited sample size (*n* = 4). For corresponding data distributions and sample sizes, see Supplementary Fig. 1.

**Fig. 4:**
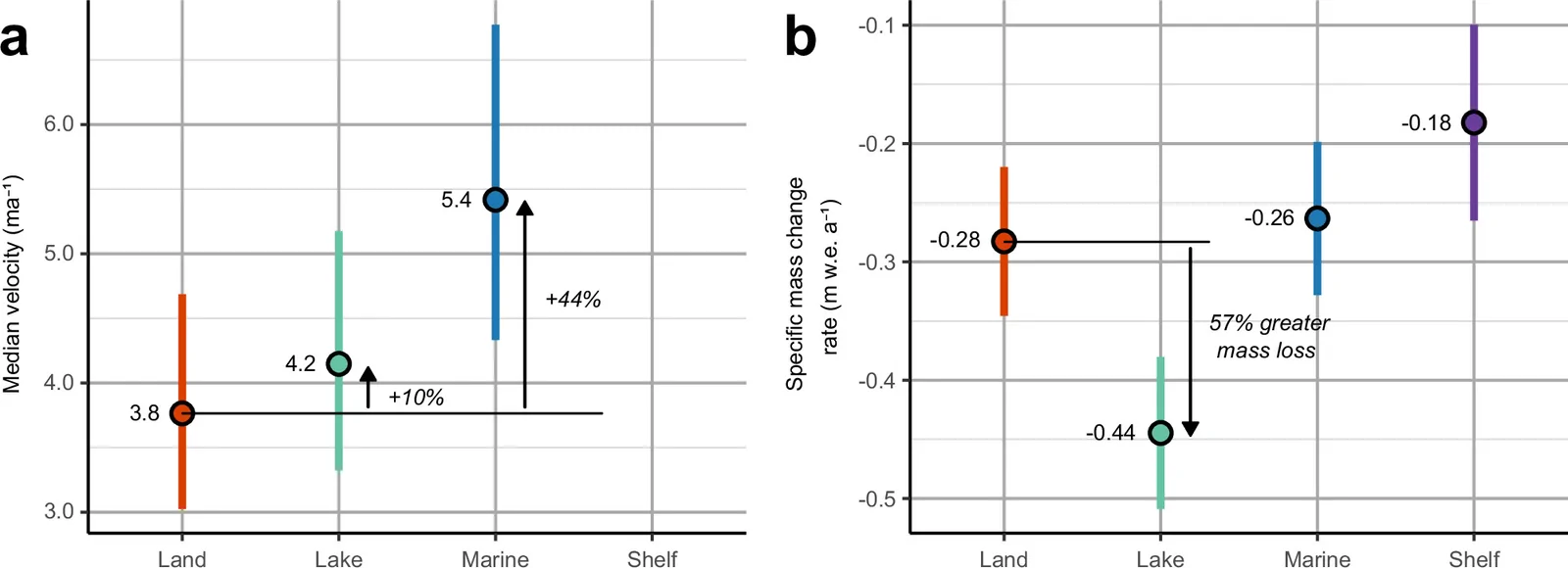
Predicted glacier dynamics by terminus type. Glacier velocities (**a**) and specific mass change rates (**b**) predicted from linear mixed-effects models. Values represent the expected mean response of a glacier in each terminus environment with other model covariates set to their median values (see Supplementary Tables 4 and 5 for covariates and full model outputs). Error bars indicate 95% confidence intervals for the means. Shelf-terminating glaciers are omitted from (**a**) due to insufficient velocity data to fit the model.

Linear mixed-effects statistical models were applied to isolate associations between terminus type and glacier dynamics whilst controlling for other key climatic, geometric and topographic influences (see “Methods”). When accounting for climate (temperature, precipitation), morphometry (ice-thickness and slope) and elevation, lake- and marine-terminating glaciers were found to flow significantly faster than land-terminating glaciers, by 10% (10^0.042^) and 44% (10^0.158^), respectively (Fig. 4a and Supplementary Table 4). When controlling for climate and a range of geometric and topographic factors (including elevation, area, debris cover and polar deviation), lake-terminating glaciers exhibited significantly greater specific mass loss than land-terminating glaciers (by −0.162 m w.e. a^−1^, i.e., 57% greater losses than at land-termini), whereas marine- and shelf-terminating glaciers lost significantly less mass than land-terminating glaciers (by 0.019 and 0.100 m w.e. a^−1^, respectively) (Fig. 4b and Supplementary Table 5).

## Discussion

Our global classification of glacier terminus types shows that, despite their low abundance, non-land termini have the potential to exert a disproportionate influence on glacier dynamics and evolution, as they collectively account for more than half of global glacier area and mass loss (Fig. 1). However, the relative importance of lake-, marine- and shelf-termini varies markedly by region (Fig. 2 and Supplementary Tables 2, 3). We also present evidence that terminus types are associated with different velocity regimes, with lake- and marine-terminating glaciers flowing, on average, 10% and 44% faster, respectively, than their land-terminating counterparts (Fig. 3a). Notably, land- and marine-terminating glaciers have highly divergent physical properties (Table 1 and Supplementary Fig. 1) yet exhibit almost identical specific mass change rates (Fig. 3b), whereas lake-terminating glaciers experience considerably greater losses (on average 57% greater than at land-termini). Importantly, both these patterns persist after controlling for other key physical and climatic drivers of glacier dynamics (Fig. 4), suggesting that these differences are independently associated with terminus type. We also note that our lake-terminating category intentionally includes all glaciers with terminus lakes, regardless of the degree of lake contact. Glaciers with limited lake interaction may therefore exhibit dynamics closer to land-terminating glaciers, suggesting our reported differences in dynamics between lake- and land-terminating glaciers are likely conservative estimates. Global patterns of glacier dynamics are broadly consistent across regions (Fig. 3c, d), with deviations largely reflecting the scarcity of a particular terminus type within a region and/or limited data availability for that terminus type (particularly in the North Asia and the Caucasus and Middle East regions).

Environmental controls at marine-terminating glaciers likely to enhance glacier velocity include: (i) reduced back pressure (resistive stress) from calving; (ii) subaqueous melting, which can destabilise a glacier terminus and promote faster flow; and (iii) deep water, which allows thicker ice and higher driving stresses^37^. Additionally, some marine-terminating glaciers experience (iv) dynamic instability, where terminus retreat over a reverse-gradient (retrograde) slope drives enhanced glacier flow that can become self-sustaining^38^. Collectively, these controls likely contribute to the enhanced velocities at marine-terminating glaciers observed in our global dataset (Fig. 3a, c) and reported regionally^29,39^. The first two of these controls also apply to lake-terminating glaciers and probably account for the more moderate velocity enhancements relative to land-terminating glaciers observed here at the global scale (Fig. 3a, c), which is consistent with regional observations from both the Himalaya^17,27,40–42^ and Greenland^43,44^.

The elevated specific mass loss rates observed at lake- compared to land-terminating glaciers in our global dataset (Fig. 3b, d) are consistent with regional observations from the Himalaya^28,45^ and New Zealand^46^, and may be supported by thermo-mechanical lake - glacier interactions^16,47^. These interactions include: (i) a thermal heat flux delivered by lakes to the ice front, promoting subaqueous ablation and undercut-driven calving; (ii) uplift and floatation of the terminus, promoting buoyancy-driven calving; (iii) propagation of lake water beneath and into an ice front, reducing basal friction; and (iv) all the above acting to increase ice surface slope and driving stress^11,47,48^. In addition, proglacial lakes may respond rapidly to atmospheric forcing due to their relatively small volume and limited water exchange, potentially allowing enhanced seasonal heat storage at the glacier terminus, which could further promote frontal ablation^49–51^. The high specific mass loss rates of lake-terminating glaciers observed here also suggest greater disequilibrium relative to other glacier types, possibly in response to relatively recent lake formation and the onset of lake - glacier feedbacks^47^ or changing lake conditions including expansion and warming^50^. Quantifying the influence of individual lakes on glacier dynamics—and the role of associated lake properties including area, depth, temperature, persistence and lake-ice interface length—represents an important direction for future research.

Additionally, or perhaps alternatively, elevated specific mass losses at lake-terminating glaciers could be explained by their particular geometries, tending to be larger and with reduced ice surface slopes in their lowermost regions compared to land-terminating glaciers (Table 1 and Supplementary Fig. 1h). The latter may also precondition the formation of proglacial lakes. Generally, the geometry of lake-terminating glaciers is likely to lead to relatively long response times^52^, which in turn favour higher specific mass loss (e.g. ref. ^45^). Land- and marine-terminating glaciers exhibit similar specific mass loss rates (Fig. 3b), but tidewater effects generally result in higher rates of frontal ice ablation at marine- compared to lake-terminating glaciers (e.g. ref. ^53^). We hypothesise that the more efficient frontal ablation process at marine-terminating glaciers allows a faster geometric adjustment to shifts in climatic forcing and therefore relatively limited specific mass loss rates. It is also possible that terminus type partly reflects underlying glacier dynamics (with larger, faster-flowing systems more likely to reach marine settings). Distinguishing the relative influence of environmental and geometric controls on glacier dynamics thus remains challenging when using glacier-wide metrics. Incorporating terminus-specific geometries (e.g., ref. ^54^) and equivalent dynamics data represents an important next step toward resolving these influences.

The patterns and magnitudes of glacier velocity and specific mass loss presented here are important because many glaciers worldwide are transitioning between terminus types. For example, marine-terminating glaciers are retreating onto land across the Arctic^35,36,55^, while the global increase in glacier lakes^33,34,56^ is causing many land-terminating glaciers to become lake-terminating (e.g., refs. ^46,57,58^). Our results suggest that such transitions will have contrasting dynamic outcomes: marine-terminating glaciers retreating onto land are likely to experience marked reductions in velocity, whereas newly lake-terminating glaciers are likely to exhibit moderate velocity increases, but substantially greater specific mass losses (Fig. 4). Consequently, continued increases in glacier lake abundance could enhance overall global rates of glacier mass loss. Furthermore, because more than half of global glacier area and mass loss can be attributed to glaciers capable of non-linear responses to atmospheric warming (Fig. 1), it is essential that such feedbacks are appropriately and explicitly incorporated into glacier evolution models, particularly those applied to individual glaciers and regional assessments where non-land termini predominate (e.g., Fig. 2 and Supplementary Table 2).

Here we have shown that glacier terminus types are associated with distinct velocity and specific mass change regimes at a global scale, and that these differences persist after accounting for other key drivers of glacier dynamics. Furthermore, non-land glacier termini, which have the potential to respond non-linearly to atmospheric warming, collectively account for more than half of global glacier area and mass loss. Our results and accompanying terminus inventory provide a foundation for refined global glacier evolution modelling, supporting improved projections of sea-level rise, regional water resources, and natural hazard risk. Crucially, they also demonstrate the need for intensified monitoring and representation of non-land glacier termini in such models, particularly as ongoing atmospheric and oceanic warming increases the likelihood of glaciers transitioning between terminus types. Future work combining observations of frontal ice loss mechanisms at non-land termini with advances in process-based modelling will be critical for capturing these dynamics and assessing their wider impacts.

## Methods

### Glacier and climate database

This study builds on the global glacier and climate database presented in Mallalieu et al.^59^. Full details of the source data are outlined in the respective paper and summarised here. Glacier locations and geometries were taken from RGI 6.0^20^. Mean annual air temperature and total annual precipitation (1991–2020) were calculated for each glacier centroid in RGI 6.0 using ECMWF ERA5 reanalysis data, with 98.4% of values from the ERA5-Land product (0.1° × 0.1° grids; ~9 km resolution) and the remainder from the ERA5-Global product (0.25° × 0.25° grids; ~30 km resolution)^60^. Median glacier surface velocity and ice thickness (2017–2018) were calculated by clipping global raster maps from Millan et al.^23^ (posted at 50 m spacing, with velocity accuracies of ≤ 10 m a^−1^ and thickness uncertainties of ~30%) to RGI 6.0 glacier outlines and applying zonal statistics in QGIS v.3.28.12. Velocities for Peripheral Glaciers and Ice Caps (PGICs) in Greenland were calculated in the same manner using the averaged 2017–2018 MEaSUREs surface velocity mosaics (posted at 200 m spacing, with accuracies of less than 5 m a^−1^)^61^. Median velocity and/or thickness values were excluded where glacier outlines overlaid fewer than three pixels to minimise the influence of outlying values (affecting 10,040 glaciers). The Mallalieu et al.^59^ database was supplemented in this study with mass change and specific mass change (i.e., mass change normalised by area) rates (2000–2019) for individual RGI 6.0 glaciers using Hugonnet et al.^5^, excluding the Caucasus and Middle East (RGI region 12) due to a lack of data corresponding to RGI 6.0 glacier outlines. Supraglacial debris-cover extents (2015–2017) were also obtained from Scherler et al.^62^ (Sentinel 2 NDSI classification) and converted to percentages by dividing by the respective RGI 6.0 glacier areas. Finally, glacier aspects from RGI 6.0 were converted to a polar deviation metric (0–180°), where higher values indicate greater deviation from a pole-facing orientation in either hemisphere. Although a newer RGI release (7.0) is available^21^, RGI 6.0 was retained to ensure compatibility with key datasets used in the wider analysis (e.g., refs. ^5,23^), which are modelled and referenced to RGI 6.0 glacier outlines.

### Glacier terminus classification

To ensure consistency and integration with the global glacier products described above, our classification of glacier termini is based on RGI 6.0 glacier outlines and attributes, which are primarily derived from Landsat TM/ETM+ imagery spanning 2000–2010^20,63^. The existing glacier terminus attribute in RGI 6.0 (*TermType*) classifies glaciers as predominantly land-, marine- or shelf-terminating, with lake-terminating glaciers identified as such in only 3 of the 19 RGI regions (Alaska, the Southern Andes, and the Antarctic and Subantarctic). Here, we retain the land, marine and shelf classifications of RGI 6.0 and create a new global-scale classification of lake-terminating glaciers to enable comparison of glacier properties and dynamics across the major terminus types. Lake-termini in all RGI regions, except Svalbard and the Antarctic and Subantarctic, are classified using the following methodology.

To identify lake-terminating RGI 6.0 glaciers, we used the global glacier lake inventory developed by Shugar et al.^33^, which was derived from modified band-ratio and thresholding analyses of 254,795 Landsat TM/ETM+/OLI scenes spanning 1990–2018. Specifically, Shugar et al.^33^ produced multi-year mosaics of glacial lakes 0.05–200 km^2^ in area and within a 1 km buffer of RGI 6.0 glacier outlines. Mapping covered all RGI regions except Svalbard and the Antarctic and Subantarctic, with reduced coverage in the Russian Arctic. In this study, we merged and dissolved lake outlines from four multi-year epochs spanning 2000–2018 to identify the presence and maximum extent of lakes during a period broadly coincident with the RGI glacier outlines (primarily 2000–2010), velocity and thickness data (2017–2018), and mass change data (2000–2019), thereby providing a temporal framework for our analysis. In addition to this temporal alignment, the Shugar et al.^33^ inventory was preferred over alternative global glacier lake inventories (e.g., ref. ^34^) because multi-year mosaics are less susceptible to lake omission when mapping over shorter timeframes (e.g., resulting from ephemeral lake drainage events, cloud- or ice-cover, or topographic shading).

**Fig. 5:**
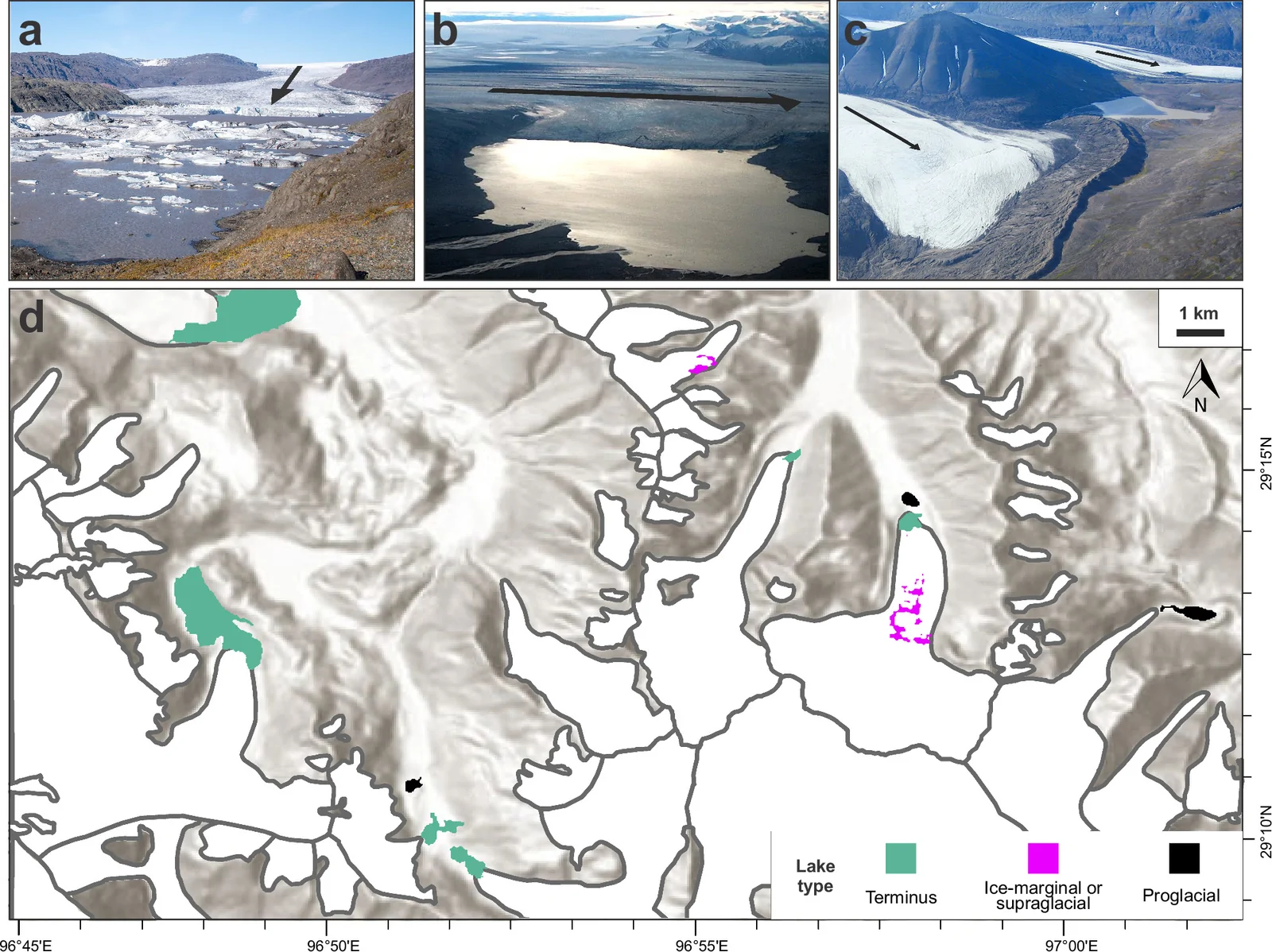
Illustration of glacial lake definitions and classifications adopted in this study. **a** Terminus lake: adjacent to and in contact with the glacier terminus (Hoffellsjökull, Iceland). **b** Ice-marginal/supraglacial lake: adjacent to and in contact with a lateral glacier margin (Skeiðarárjökull, Iceland). **c** Proglacial lake: near, but not in contact with, a glacier terminus (unnamed glaciers, Svalbard). **d** Example of our classification applied to the Shugar et al.^33^ global glacial lake dataset, illustrating terminus, ice-marginal/supraglacial, and proglacial lakes in southeastern Tibet. Only glaciers with ‘terminus lakes’ are considered lake-terminating in this analysis. Lake polygons in (**d**) represent maximum lake extent over the period 2000-2018. Black arrows in (**a–c**) indicate the direction of glacier flow. RGI 6.0 glacier outlines from RGI Consortium^20^. Image **b** courtesy of Fiona Tweed.

Dissolved lake outlines were filtered to create three lake categories: terminus, ice-marginal/supraglacial and proglacial. Here we define terminus lakes as those located adjacent to and in contact with a glacier terminus; ice-marginal/supraglacial lakes as those adjacent to and in contact with a lateral glacier margin or entirely supraglacial; and proglacial lakes as those near to, but not in contact with, a glacier terminus (Fig. 5a–c). Proglacial status was assigned to all lakes lying entirely beyond a 60 m buffer applied to the RGI 6.0 glacier outlines. The 60 m buffer was chosen to accommodate a potential one Landsat pixel (30 m) misclassification in both the glacier and lake outlines. An elevation filter was applied to the remaining lakes to identify those more than 100 m above the minimum glacier elevation (using the respective lake and glacier attributes), which were assigned ice-marginal/supraglacial status. All remaining lakes were assigned terminus status (Fig. 5d). Finally, any RGI 6.0 glacier outline intersecting within 60 m of a terminus lake was classified as lake-terminating. Thus, for the purposes of our analysis, RGI 6.0 glaciers intersecting terminus lakes at any point in the period 2000–2018 (except those in Svalbard and the Antarctic and Subantarctic) are considered lake-terminating. To maintain consistency and replicability, no manual adjustments were made to the classification, except for three glaciers in the Southern Andes (RGI60−17.00172, RGI60−17.04928, and RGI60−17.05184), which were manually assigned lake-terminating status. These glaciers drain into large lakes (Lago Argentino and Lago San Martín), excluded from the Shugar et al.^33^ inventory because their areas exceeded the 200 km^2^ upper limit for mapping.

Lake-termini in Svalbard were classified using the 2008–2012 glacier lake inventory in Wieczorek et al.^64^, based on manual digitisation of lakes from high-resolution (0.5 m) aerial imagery. For consistency with the global classification, terminus lakes were determined by removing lake outlines less than 0.05 km^2^ in area and applying the same spatial and elevational filters outlined above for the Shugar et al.^33^ inventory. Svalbard terminus lake elevations were determined using Shuttle Radar Topography Mission (SRTM) data^65^. Due to a lack of alternative sources, our classification retains the lake-terminating status of glaciers in the Antarctic and Subantarctic region directly from RGI 6.0^20^. In our final database, any glaciers classified as marine-terminating in RGI 6.0, but also intersecting a terminus lake in our classification, retain their marine-terminating status. The global and regional descriptive statistics presented in Figs. 1, 2 and Supplementary Tables 1–3 are derived from this globally complete terminus classification. Mass change rate (Gt a^−1^) is used in this section of the analysis to illustrate the global contributions of different terminus types to sea-level rise.

### Classification validation

Direct comparison of our classification with the RGI 7.0 lake-terminating glacier inventory by Steiner et al.^66^ is complicated by substantial revisions between RGI 6.0 and 7.0 (including changes to 73% of glacier outlines and a 27% increase in glacier count, though only a 0.1% increase in total glacier area^67^). Absolute glacier numbers are therefore not directly comparable, but percentage- and area-based metrics remain meaningful. Steiner et al.^66^ present three sub-categories of lake-terminating glaciers; for comparability, we derive values from categories 1–3 combined (all glaciers with a terminus lake) and categories 2–3 combined (only glaciers considered by Steiner et al.^66^ likely to be dynamically impacted by their lakes, and thus retained as lake-terminating in RGI 7.0). Our global estimates are more conservative than categories 1–3 combined (Table 2), likely reflecting our more restrictive minimum lake size threshold for determining lake-terminating status (≥0.05 km^2^ versus ≥0.01 km^2^). However, our estimates are in good agreement with categories 2–3 combined (Table 2), our slightly higher values likely reflecting the longer timeframe over which glaciers could be classified as lake-terminating using the multi-temporal Shugar et al.^33^ inventory (in contrast, Steiner et al.^66^ used satellite images and lake inventories dated within ±2 years and ±10 years of 2000, respectively). Compared to the available regional lake-termini data in RGI 6.0^20^, our classification identifies 68% and 174% more lake-terminating glaciers in Alaska and the Southern Andes, respectively. However, our percentage values of lake-termini in Alaska (0.9%) and the Southern Andes (2.3%) are broadly comparable to those reported by Steiner et al.^66^ (0.7% and 1.9%, respectively, for categories 2–3 combined), suggesting that the original RGI 6.0 classification considerably underreported the number of lake-termini in these regions. In our classification, lake-termini are potentially underrepresented in the Russian Arctic RGI region due to a lack of suitable imagery for Franz Josef Land in the Shugar et al.^33^ lake inventory.

**Table 2:** Comparison of global lake-terminating glacier statistics between this study and Steiner et al.^66^

	This study: RGI 6.0 lake-terminating glaciers	Steiner et al.^66^: RGI 7.0 lake-terminating glaciers	
		Categories 2 and 3	Categories 1, 2 and 3
Global glaciers terminating in a lake (%)	1.6	1.4	1.9
Global glacier area terminating in a lake (%)	11.5	11.4	15.7
Global glacier area terminating in a lake (10^3 ^km^2^)	86.0	80.8	111.4

Values for Steiner et al.^66^ represent the sum of the indicated categories.

### Exploring variability in glacier dynamics across terminus types

To investigate how terminus type relates to glacier velocity and specific mass change, our database was standardised by excluding glaciers with spurious geometries and/or dynamic properties likely to be unrepresentative of local climate and topography. This included: 461 glaciers with estimated geometries in RGI 6.0; 955 PGICs strongly connected to the Greenland Ice Sheet^20^; 1343 glaciers identified as observed, possible, or probable surge-type (comprising 0.6% of land-, 1.7% of lake-, and 3.8% of marine-termini respectively)^68^; and 2729 glaciers within 5 km of a volcano^69^ which may be affected by enhanced ice velocities^59,70^ and elevation anomalies^71^. Of the 216,502 glaciers in RGI 6.0, our final standardised database returned velocity and specific mass change rate values for 83.9% (*n* = 181,603) and 93.9% (*n* = 203,372), respectively. Specific (i.e., area-normalised) mass change rate (m w.e. a^−1^) is used in this section of the analysis to facilitate comparison across terminus types. All descriptive and inferential statistics presented in Table 1, Figs. 3, 4, Supplementary Fig. 1 and Supplementary Tables 4, 5 are derived from this standardised dataset. Confidence intervals for median values in Table 1 were estimated using non-parametric bootstrapping (500 resamples; percentile method).

To further quantify associations between terminus type and glacier dynamics, while controlling for other key climatic, geometric and topographic influences, linear mixed-effects models (LMMs) were implemented in R v.4.2.1 using the lme4 package^72^. The first LMM (Supplementary Table 4) was fitted with median glacier velocity as the response variable using a log_10-_transformation to normalise its inverse Gaussian distribution. Continuous covariates were scaled, and all covariates were screened for multicollinearity using Pearson correlation coefficients or variance inflation factors prior to model fitting. Strong collinearity (|r| > ±0.7^73^) was found between median glacier thickness and slope (*r* = −0.89), and between mean annual air temperature and total annual precipitation (*r* = 0.96). To retain all relevant covariates without redundancy, principal component analysis (PCA) was used to derive two composite variables: a glacier morphometry PC (based on slope and Box-Cox-transformed thickness) and a climate PC (based on temperature and log_10_-transformed precipitation). The glacier morphometry PC explained 74% of the variance in thickness and slope, with higher values representing thicker, less steep glaciers. The climate PC explained 92% of the variance in temperature and precipitation, with higher values representing warmer, wetter climates. The velocity LMM was parameterised: log_10_(median glacier velocity) = β0 + (β1 × glacier terminus type) + (β2 × morphometry PC) + (β3 × median glacier elevation) + (β4 × climate PC) + b_RGI REGION_; where β0 is the intercept, β1–β4 are fixed-effect coefficients, and b_RGI REGION_ is a random intercept for each RGI 6.0 region. Glacier terminus type was included as a categorical covariate indicating whether a glacier terminated in a land, lake or marine setting, with land set as the reference level. Shelf-terminating glaciers were excluded due to insufficient velocity data (*n* = 4). Median elevation was included to account for the contrast in elevations between glaciers terminating in marine and other environments.

The second LMM (Supplementary Table 5) followed the same framework, using specific mass change rate (2000–2019) as the response variable. To address a strongly skewed and leptokurtic distribution, specific mass change rates more than 3 standard deviations from the mean were excluded (*n* = 1742). The specific mass change rate LMM was parameterised: specific mass change rate = β0 + (β1 × glacier terminus type) + (β2 × glacier elevation range) + (β3 × median glacier elevation) + (β4 × glacier area) + (β5 × debris cover) + (β6 × polar deviation) + (β7 × climate PC) + b_RGI REGION_; where β0 is the intercept, β1–β7 are fixed-effect coefficients, and b_RGI REGION_ is a random intercept for each RGI 6.0 region. Glacier terminus type was included as a categorical covariate (land, lake, marine or shelf), with land set as the reference level. A variance function (varExp in the nlme package^74^) was included to account for heteroscedasticity by modelling residual variance as an exponential function of the fitted values. Diagnostics for both models confirmed residual normality, homoscedasticity and independence. *P*-values for fixed-effects were obtained using the lmerTest package^75^.

## Supplementary information

Supplementary Information

Transparent Peer Review file

## Data Availability

The database of global glacier and climate properties (including terminus inventory) generated in this study is available from Zenodo (https://doi.org/10.5281/zenodo.22712528). The underlying data used in this study are available from the following sources: RGI 6.0 glacier polygons and properties^20^ (https://doi.org/10.7265/4m1f-gd79); global glacier velocity and thickness maps^23^ (https://doi.org/10.6096/1007); Greenland glacier velocity mosaics^61^ (https://doi.org/10.5067/RS8GFZ848ZU9); glacier mass change data^5^ (10.6096/13); glacier debris cover data^62^ (https://doi.org/10.5880/GFZ.3.3.2018.005); global glacier lake polygons^33^ (https://doi.org/10.5067/UO20NYM3YQB4); Svalbard glacier lake polygons^64^ (https://doi.org/10.1017/jog.2023.34 - in supplementary materials); glacier proximity to volcano data^69^ (https://doi.org/10.1016/j.gloplacha.2020.103356 - in supplementary data). ECMWF ERA5 reanalysis climate data^60^ can be accessed at https://www.ecmwf.int/en/forecasts/dataset/ecmwf-reanalysis-v5.

## Code availability

The R code used for the data processing, statistical analyses and associated figures presented in this study is available from GitHub (https://github.com/joemallalieu/global_glacier_termini_and_dynamics) and archived on Zenodo (https://doi.org/10.5281/zenodo.22738485).
