# Supplementary material for: Global-scale variability of glacier velocity and mass loss across terminus environments
Source: Nat Commun. 2026 Oct 23;17:10873. doi: 10.1038/s41467-026-78654-y (PMC13694695; doi:10.1038/s41467-026-78654-y)
Supplement: Supplementary file 1 [file 41467_2026_78654_MOESM1_ESM.pdf]

# Global-scale variability of glacier velocity and mass loss across terminus environments

**Joseph Mallalieu[1,2,3*], Jonathan L. Carrivick[2], Matthias Huss[4,5], Iestyn D. Barr[6], Nicholas E. Barrand[1], Cassandra L. Raby[3]**

[1]School of Geography, Earth and Environmental Sciences, University of Birmingham, Birmingham, UK

[2]School of Geography and water@leeds, University of Leeds, Leeds, UK

[3]School of Biology, University of Leeds, Leeds, UK

[4]Laboratory of Hydraulics, Hydrology and Glaciology (VAW), ETH Zurich, Zurich, Switzerland

[5]Swiss Federal Institute for Forest, Snow and Landscape Research (WSL), Sion, Switzerland

[6]Department of Natural Sciences, Manchester Metropolitan University, Manchester, UK

*Corresponding author: j.mallalieu@bham.ac.uk

**SUPPLEMENTARY INFORMATION**

Contents: Supplementary Tables 1-5; Supplementary Figure 1.

| RGI region | Number of glaciers by terminus type | | | | Percentage of glaciers by terminus type | | | |
|---|---|---|---|---|---|---|---|---|
| | Land | Lake | Marine | Shelf | Land | Lake | Marine | Shelf |
| Alaska | 26808 | 249 | 51 | 0 | 98.9 | 0.9 | 0.2 | 0.0 |
| W. Canada & USA | 18520 | 335 | 0 | 0 | 98.2 | 1.8 | 0.0 | 0.0 |
| Arctic Canada North | 4213 | 85 | 258 | 0 | 92.5 | 1.9 | 5.7 | 0.0 |
| Arctic Canada South | 6999 | 348 | 68 | 0 | 94.4 | 4.7 | 0.9 | 0.0 |
| Greenland Periphery | 18604 | 754 | 903 | 0 | 91.8 | 3.7 | 4.5 | 0.0 |
| Iceland | 527 | 41 | 0 | 0 | 92.8 | 7.2 | 0.0 | 0.0 |
| Svalbard & Jan Mayen | 1408 | 66 | 141 | 0 | 87.2 | 4.1 | 8.7 | 0.0 |
| Scandinavia | 3120 | 297 | 0 | 0 | 91.3 | 8.7 | 0.0 | 0.0 |
| Russian Arctic | 587 | 103 | 375 | 4 | 54.9 | 9.6 | 35.1 | 0.4 |
| North Asia | 5114 | 35 | 2 | 0 | 99.3 | 0.7 | 0.0 | 0.0 |
| Central Europe | 3907 | 20 | 0 | 0 | 99.5 | 0.5 | 0.0 | 0.0 |
| Caucasus & Middle East | 1885 | 3 | 0 | 0 | 99.8 | 0.2 | 0.0 | 0.0 |
| Central Asia | 54124 | 305 | 0 | 0 | 99.4 | 0.6 | 0.0 | 0.0 |
| South Asia West | 27879 | 109 | 0 | 0 | 99.6 | 0.4 | 0.0 | 0.0 |
| South Asia East | 12814 | 305 | 0 | 0 | 97.7 | 2.3 | 0.0 | 0.0 |
| Low Latitudes | 2855 | 84 | 0 | 0 | 97.1 | 2.9 | 0.0 | 0.0 |
| Southern Andes | 15458 | 365 | 85 | 0 | 97.2 | 2.3 | 0.5 | 0.0 |
| New Zealand | 3510 | 27 | 0 | 0 | 99.2 | 0.8 | 0.0 | 0.0 |
| Antarctic & Subantarctic | 1231 | 17 | 1339 | 165 | 44.7 | 0.6 | 48.7 | 6.0 |

**Supplementary Table 1. Number and percentage of glaciers terminating in land-, lake-, marine-, or shelf-environments in each of the RGI subregions.** Glacier numbers from RGI Consortium[1].

| RGI region | Total glacier area (km$^2$) by terminus type | | | | Percentage of glacier area by terminus type | | | |
|---|---|---|---|---|---|---|---|---|
| | Land | Lake | Marine | Shelf | Land | Lake | Marine | Shelf |
| Alaska | 51530 | 23545 | 11650 | 0 | 59.4 | 27.2 | 13.4 | 0.0 |
| W. Canada & USA | 12636 | 1889 | 0 | 0 | 87.0 | 13.0 | 0.0 | 0.0 |
| Arctic Canada North | 50302 | 5698 | 49111 | 0 | 47.9 | 5.4 | 46.7 | 0.0 |
| Arctic Canada South | 27611 | 10248 | 3030 | 0 | 67.5 | 25.1 | 7.4 | 0.0 |
| Greenland Periphery | 54764 | 10332 | 64975 | 0 | 42.1 | 7.9 | 50.0 | 0.0 |
| Iceland | 3865 | 7194 | 0 | 0 | 35.0 | 65.1 | 0.0 | 0.0 |
| Svalbard & Jan Mayen | 16061 | 3008 | 14890 | 0 | 47.3 | 8.9 | 43.9 | 0.0 |
| Scandinavia | 2313 | 636 | 0 | 0 | 78.4 | 21.6 | 0.0 | 0.0 |
| Russian Arctic | 11436 | 6722 | 32615 | 819 | 22.2 | 13.0 | 63.2 | 1.6 |
| North Asia | 2404 | 4 | 3 | 0 | 99.7 | 0.2 | 0.1 | 0.0 |
| Central Europe | 2007 | 85 | 0 | 0 | 95.9 | 4.1 | 0.0 | 0.0 |
| Caucasus & Middle East | 1282 | 25 | 0 | 0 | 98.1 | 1.9 | 0.0 | 0.0 |
| Central Asia | 46971 | 2333 | 0 | 0 | 95.3 | 4.7 | 0.0 | 0.0 |
| South Asia West | 33077 | 492 | 0 | 0 | 98.5 | 1.5 | 0.0 | 0.0 |
| South Asia East | 12974 | 1760 | 0 | 0 | 88.1 | 12.0 | 0.0 | 0.0 |
| Low Latitudes | 2057 | 284 | 0 | 0 | 87.9 | 12.1 | 0.0 | 0.0 |
| Southern Andes | 11239 | 11133 | 7057 | 0 | 38.2 | 37.8 | 24.0 | 0.0 |
| New Zealand | 914 | 248 | 0 | 0 | 78.7 | 21.3 | 0.0 | 0.0 |
| Antarctic & Subantarctic | 1360 | 316 | 48053 | 83138 | 1.0 | 0.2 | 36.2 | 62.6 |

**Supplementary Table 2. Cumulative and percentage area of glaciers terminating in land-, lake-, marine-, or shelf-environments in each of the RGI subregions.** Glacier areas from RGI Consortium[1].

| RGI region | Mass change rate 2000-2019 (Gt a$^{-1}$) | | | | Percentage of mass change rate 2000-2019 by terminus type | | | |
|---|---|---|---|---|---|---|---|---|
| | Land | Lake | Marine | Shelf | Land | Lake | Marine | Shelf |
| Alaska | -35.28 | -23.58 | -7.77 | NA | 53.0 | 35.4 | 11.7 | NA |
| W. Canada & USA | -5.98 | -1.56 | NA | NA | 79.3 | 20.7 | NA | NA |
| Arctic Canada North | -16.83 | -1.80 | -11.93 | NA | 55.1 | 5.9 | 39.0 | NA |
| Arctic Canada South | -17.16 | -7.74 | -1.60 | NA | 64.8 | 29.2 | 6.0 | NA |
| Greenland Periphery | -19.55 | -4.30 | -11.66 | NA | 55.1 | 12.1 | 32.8 | NA |
| Iceland | -3.01 | -6.35 | NA | NA | 32.2 | 67.8 | NA | NA |
| Svalbard & Jan Mayen | -3.71 | -0.69 | -6.14 | NA | 35.2 | 6.6 | 58.3 | NA |
| Scandinavia | -1.24 | -0.43 | NA | NA | 74.3 | 25.8 | NA | NA |
| Russian Arctic | -1.11 | -0.94 | -8.22 | -0.14 | 10.7 | 9.0 | 79.0 | 1.3 |
| North Asia | -1.21 | 0.00 | 0.00 | NA | 100.0 | 0.0 | 0.0 | NA |
| Central Europe | -1.59 | -0.09 | NA | NA | 94.6 | 5.4 | NA | NA |
| Central Asia | -8.63 | -0.97 | NA | NA | 89.9 | 10.1 | NA | NA |
| South Asia West | -4.40 | -0.15 | NA | NA | 96.7 | 3.3 | NA | NA |
| South Asia East | -5.69 | -1.18 | NA | NA | 82.8 | 17.2 | NA | NA |
| Low Latitudes | -0.79 | -0.14 | NA | NA | 85.0 | 15.1 | NA | NA |
| Southern Andes | -4.10 | -13.10 | -3.41 | NA | 19.9 | 63.6 | 16.6 | NA |
| New Zealand | -0.38 | -0.25 | NA | NA | 60.3 | 39.7 | NA | NA |
| Antarctic & Subantarctic | -0.56 | -0.48 | -9.69 | -10.08 | 2.7 | 2.3 | 46.6 | 48.4 |

**Supplementary Table 3. Cumulative and percentage mass change rate (2000-2019) of glaciers terminating in land-, lake-, marine-, or shelf-environments in each of the RGI subregions.** NB. The Caucasus and Middle East RGI region is omitted due to the absence of mass change rate data corresponding to RGI 6.0 glacier outlines. Mass change data from Hugonnet et al.[2].

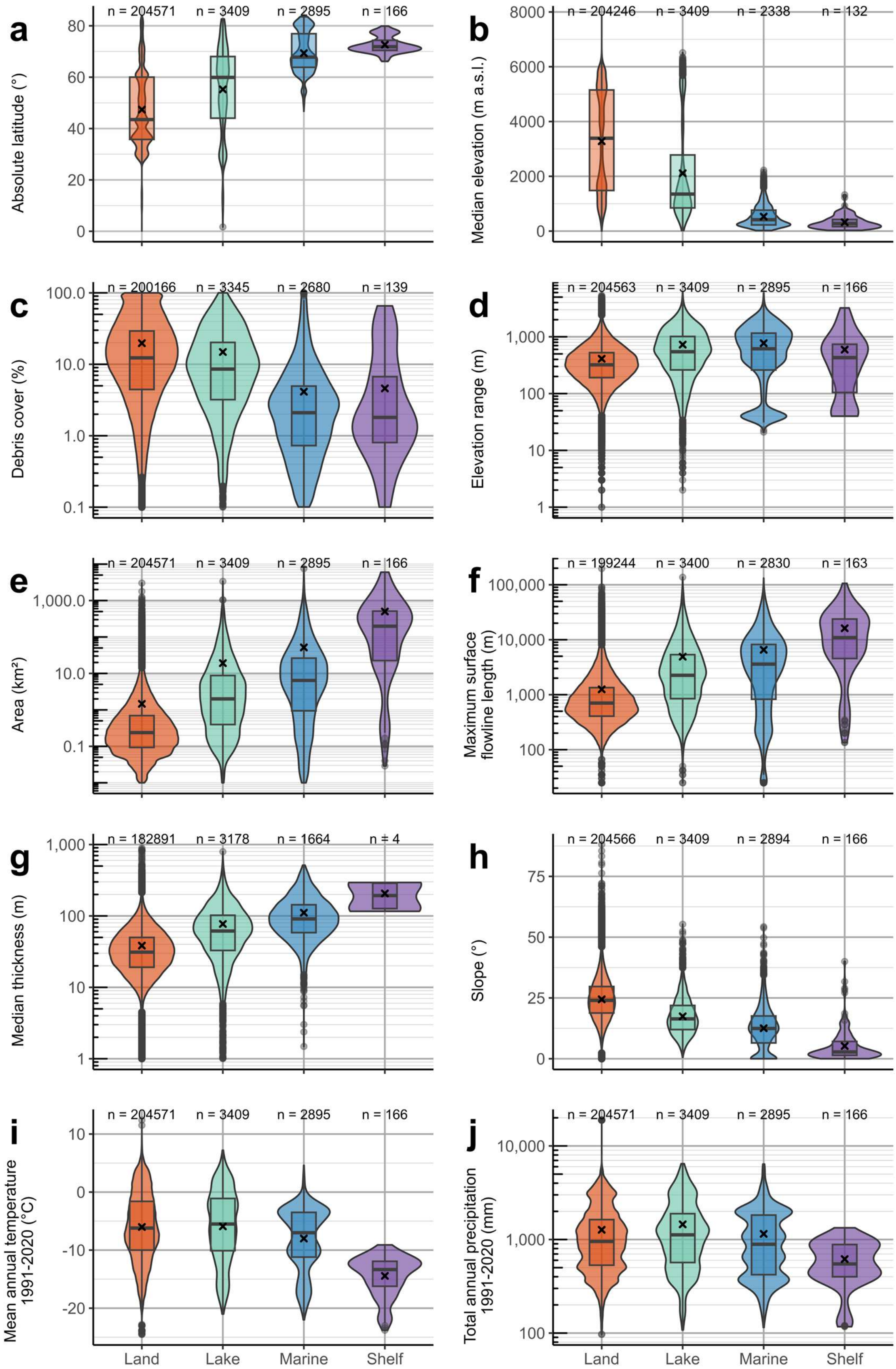


**Supplementary Fig. 1. Glacier properties by terminus type.** Global distributions of key glacier attributes across land-, lake-, marine-, and shelf-terminating glaciers: absolute latitude (**a**), median elevation (**b**), debris cover (**c**), elevation range (**d**), area (**e**), maximum surface flowline length (**f**), median thickness (**g**), slope (**h**), mean annual temperature (1991-2020) (**i**), and total annual precipitation (1991-2020) (**j**). Violin plots show data distributions, black crosses represent mean values, and box plots show median values and interquartile ranges. To improve clarity, the y-axes in (**c**) and (**g**) are cropped, omitting 18,653 (9.0%) and 2,555 (1.4%) outlying values respectively.

| Independent variable | Estimate | 95% Confidence level | | Std. error | t value | p value |
|---|---|---|---|---|---|---|
| | | lower | upper | | | |
| (Intercept) | 0.612 | 0.515 | 0.709 | 0.048 | 12.626 | <0.001 |
| Glacier terminus type: lake | 0.042 | 0.028 | 0.056 | 0.007 | 5.744 | <0.001 |
| Glacier terminus type: marine | 0.158 | 0.138 | 0.178 | 0.010 | 15.521 | <0.001 |
| Glacier morphometry PC | 0.101 | 0.099 | 0.102 | 0.001 | 117.016 | <0.001 |
| Median glacier elevation | -0.128 | -0.135 | -0.122 | 0.003 | -37.718 | <0.001 |
| Climate PC | 0.027 | 0.024 | 0.029 | 0.001 | 19.073 | <0.001 |

**Supplementary Table 4. Linear mixed-effects model showing $log_{10}$-transformed median glacier velocity as a function of terminus type and independent physical and climatic covariates.** The model includes a random intercept for RGI region (n = 19). n = 175,925; $R^2m$ = 0.18, $R^2c$ = 0.38. The reference category for the glacier terminus type variable is 'land'. PC = principal component.

| Independent variable | Estimate | 95% Confidence level | | Std. error | t value | p value |
|---|---|---|---|---|---|---|
| | | lower | upper | | | |
| (Intercept) | -0.294 | -0.357 | -0.231 | 0.032 | -9.167 | <0.001 |
| Glacier terminus type: lake | -0.162 | -0.175 | -0.149 | 0.007 | -24.231 | <0.001 |
| Glacier terminus type: marine | 0.019 | 0.004 | 0.035 | 0.008 | 2.406 | 0.016 |
| Glacier terminus type: shelf | 0.100 | 0.046 | 0.154 | 0.028 | 3.646 | <0.001 |
| Glacier elevation range | -0.018 | -0.019 | -0.016 | 0.001 | -22.403 | <0.001 |
| Median glacier elevation | 0.033 | 0.029 | 0.038 | 0.002 | 13.791 | <0.001 |
| Glacier area | -0.004 | -0.006 | -0.002 | 0.001 | -4.382 | <0.001 |
| Debris cover (%) | -0.012 | -0.014 | -0.011 | 0.001 | -14.869 | <0.001 |
| Polar deviation | 0.014 | 0.013 | 0.016 | 0.001 | 19.282 | <0.001 |
| Climate PC | -0.046 | -0.048 | -0.044 | 0.001 | -43.347 | <0.001 |

**Supplementary Table 5. Linear mixed-effects model showing specific mass change rate (2000-2019) as a function of glacier terminus type and independent physical and climatic covariates.** The model includes a random intercept for RGI region (n = 19). n = 196,747; $R^2m$ = 0.08, $R^2c$ = 0.28. The reference category for the glacier terminus type variable is 'land'. PC = principal component.

**SUPPLEMENTARY REFERENCES**


1. RGI Consortium. Randolph Glacier Inventory (RGI) - A Dataset of Global Glacier Outlines: Version 6.0. NSIDC: National Snow and Ice Data Center. https://doi.org/10.7265/4m1f-gd79 (2017).
2. Hugonnet, R. *et al.* Accelerated global glacier mass loss in the early twenty-first century. *Nature* **592**, 726-731 (2021).
