# Supplementary material for: Global-scale variability of glacier velocity and mass loss across terminus environments
Source: Nat Commun. 2026 Oct 23;17:10873. doi: 10.1038/s41467-026-78654-y (PMC13694695; doi:10.1038/s41467-026-78654-y)
Supplement: Supplementary file 2 [file 41467_2026_78654_MOESM2_ESM.pdf]

nature portfolio

Peer Review File

Global-scale variability of glacier velocity and mass loss across terminus environments

Corresponding Author: Dr Joseph Mallalieu

This file contains all reviewer reports in order by version, followed by all author rebuttals in order by version.

Version 0:

Reviewer comments:

Reviewer #1

(Remarks to the Author)
Review of „Global-scale influence of terminus environment on glacier velocity and mass loss“ by Mallalieu et al.

The paper brings concise overview of the glacier terminus type and relate it to diverse glacier characteristics. It might be a useful dataset for further studies and a baseline for more developed studies.

The methods are clear and well described. I am just not sure why excluding surge type glacier when assessing glacier dynamics. Surging is part of the game and affects velocity and mass balance significantly. Perhaps it might be interesting to (at least) look at the surge type glaciers separately and provide an overview on distribution of surge type glaciers within the four categories. I guess the surge type glaciers will mostly fall in lake and certainly marine terminating glaciers category? Also, can you comment on the highest velocity of the marine terminating glaciers in Alaska (Fig 3c) – isnt it affected by surging? Is it purely climatic driven?

Another issue worth exploring (at least discussing) might be to turn the problem around – for example the large and fast flowing glaciers often reach the marine environment. So the terminus type (marine in this case) might also be a result of the high glacier dynamics, not only the cause of the high dynamics. On the contrary, small glaciers usually exhibit low dynamics and do not reach marine environment or develop ice-contact lakes. I think you mentioned similar issue while talking about the lake-terminating type and the effect of topography. Can you comment on that?

Overall, I think the paper reads well, I dont have any detailed comments to the text itself. Also I cannot comment on the language as unlike most of the authors I am not a native English language speaker.

Thanks for taking my comments/suggestions into consideration, I hope it might help!

(Remarks on code availability)

Reviewer #2

(Remarks to the Author)
The goal of this manuscript is to quantify the influence of terminus type of dynamics of mountain glaciers, namely velocity and mass change. To do this, the authors categorize mountain glaciers defined by the Randolph Glacier Inventory (RGI) version 6.0 as land-, lake-, or marine-terminating. The main findings are that lake- and marine-terminating glaciers flow faster than land-terminating glaciers (not that surprising) and that lake-terminating glaciers have higher specific mass loss rates than both land- and marine-terminating glaciers (more interesting). The overall quality of the presentation is good. The manuscript is well-written and the figures and tables clear communicate the findings. That said, I do have two major concerns. The first is about the methods that the authors use to assess the impact of lakes on glaciers. The second is that approach used to separate glaciers into lake-terminating category. In my opinion these issues seriously undermine the robustness of the findings and for this reason I recommend that the manuscript is rejected.

Major comments

My first concern is that the current statistical analysis does not sufficiently support the primary goal of the study which is "to quantify… the influence of terminus type on glacier dynamics at the global scale" (L51-52). Since the authors do not directly measure the mass loss caused by ice-marginal lakes, the analysis cannot differentiate between mass loss caused by ice-marginal lakes from that caused by ice-atmosphere interactions. I think the authors are well aware of this limitation. But the problem is that the statement in the abstract that reads "…mass loss rates at lake-terminating glaciers are 57% greater than those at land- and marine-terminating glaciers" is deceptive because the analysis provides no evidence that lakes are actually responsible for these differences (even though it is strongly implied). Without a more direct attribution of glacier mass loss to processes occurring at the terminus, the conclusions of the manuscript are relatively weak e.g. "non-land termini have the potential to exert a disproportionate influence on glacier dynamics" (L152-153) and "glacier terminus types are associated with distinct velocity and specific mass change regimes" (L214-215). Unfortunately these statements are lacking the causality required to fulfill the ambitious goal of the study and contribute to scientific understanding.

My second major concern is that the classification scheme likely overestimates the number of glaciers that are actually impacted by ice-marginal lakes. The study classifies lake-terminating glaciers as those in the RGI inventory which are within 60 m of a lake identified by the global glacier lake inventory developed by Shugar et al. and no more than 100 m above the minimum glacier elevation. However, following this approach, a glacier with a small lake near the side of its terminus would be classified as lake-terminating, even if the lake has very little influence on its dynamics. Inclusion of this "lake-terminating" (but not really) glacier into the subsequent statistical analysis, is likely to skew the velocity and mass change of the lake-terminating category. Currently the authors provide no discussion of how this scenario would impact their findings. A better approach would be to determine the influence of ice-marginal lakes more directly. This is what the Steiner et al. (2025) paper does for example by looking for evidence of calving into ice-marginal lakes. I realize that modifying the analysis would be challenging at this stage but this is a major flaw of the current approach.

The authors justify the number of glaciers impacted by ice-marginal lakes by providing a coarse comparison between the percentage of lake-terminating glaciers identified by their approach relative to Steiner et al. (2025). However, they do not mention that Steiner et al. (2025) differentiates lake-terminating glaciers into three classes, the third of which contains glaciers that "do not have visible impacts due to the lake except along a very limited (<10%) portion of the terminus". For the same reason, it is perhaps not surprising that the authors identify 68% and 174% more lake-terminating glaciers in Alaska and Southern Andes (L323-324) than RGI 6.0. The problem is that the narrative of the manuscript implies that ice-marginal lakes cause glaciers to flow 10% faster and lose 57% more mass. But these values are unlikely to be accurate if the numbers (and parts) of glaciers that are impacted by lakes are dramatically overestimated.

Specific comments

L39: It would be useful to clarify somewhere in this paragraph that these glacier evolution models are for "mountain" glaciers as opposed to tidewater glaciers flowing from ice sheets which do account for ice dynamics.

L43-44: Missing from this paragraph is a couple of statements that review the known differences between dynamics of land- vs lake- vs marine-terminating glaciers.

L108-110: I don't think that these statistics are very useful since they are not normalized for area. The fact that bigger, marine-terminating glaciers flow faster than smaller land- and lake-terminating glaciers is self-evident.

L135-136: It's not clear why the volume or area of the glacier is also not accounted for.

L378-379: I don't think this conforms to the reporting standards for Nature Portfolio journals which require code and data to be available to reviewers during peer review process

References

Steiner et al. (2025) https://essd.copernicus.org/preprints/essd-2025-315/

(Remarks on code availability)
The authors did not make the code (or data) available to the reviewers.

Reviewer #3

(Remarks to the Author)
This manuscript provides a valuable and timely update to prior global synthesis studies of glacier mass and volume change estimates by examining how terminal environment plays a role in explaining variation in specific mass change. Their main findings are that marine-terminating glaciers are faster than their land-terminating counterparts, but they lose less mass than land-terminating glaciers. They also find that lake-terminating glaciers lose the most mass – even when examined regionally (for most regions). The authors claim that the reasons for this increase in mass loss for lake-terminating glaciers results from enhanced terminus-lake interaction and the unique terminus geometry of lake-terminating glaciers that permits longer response times.

I am, however, not convinced that the results presented here are at the level of Nature Communications for a few reasons.

First, the authors do not demonstrate cause and effect. They provide compelling correlations, but the physical mechanisms remain inferred, not demonstrated. Further, Table 1 shows that lake-terminating glaciers occupy warmer environments than other terminus types. Is this intrinsic to this terminus environment or a consequence of other geographic factors, like elevation and latitude, both of which strongly influence environmental temperature. The current manuscript makes it difficult to assess if the reported differences in mass loss by terminus type are simply a consequence of where these different terminus types are found.

In addition, because lakes are smaller volumes, and these glaciers are generally in warmer conditions than marine-terminating glaciers I would expect that the water temperature near the terminus of lake-terminating glaciers to be warmer than marine-terminating counterparts. Lakes, unlike fjords, can more rapidly warm and store seasonal heat and this may explain why they lose more mass. The manuscript does not adequately discuss this point.

Some smaller points –
• I'm not sure what a shelf-terminating glacier is – perhaps because these are exclusively in the Antarctic, these are glaciers that terminate in an ice shelf? I'm not sure they add much value to the manuscript – they are not discussed at length and their population is small and regional.
• 154: "they collectively account for more than half of global glacier area and mass loss (Fig 1)" – but Figure 1 does not show mass loss, just area and there is no global accounting, just regional. It would be good to provide the data on this statement, since it constitutes most of their thesis. What I'm asking for is lake-terminating glacier area/global glacier area and lake-terminating glacier mass loss/global glacier mass loss. You could make a global area pie chart for Fig. 1 for the first request and then do some calculations to provide the stats for the latter. This would allow us to see how much non-land-terminating glaciers drive the global average mass loss estimates.
• 189: reduced gradients in what?

(Remarks on code availability)

Version 1:

Reviewer comments:

Reviewer #1

(Remarks to the Author)
dear authors,

thanks for clarifying the issues raised in my previous review, I was expecting the surging to be more important. I think the article reads well now and the additions made on request of other reviewers helped as well. I guess, the manuscript is ready for publication now.

with regards,
Jan

(Remarks on code availability)

Reviewer #2

(Remarks to the Author)
I thank the authors for responding to my comments. The updated manuscript has been lightly revised. The main changes are that much of the casual language between terminus type and mass loss has been moderated. The manuscript also provides a more direct comparison with a previous study which quantified the terminus types of global glaciers e.g. Table 2 (Steiner et al., 2026).

Despite the slight reframing of the manuscript, I'm still struggling to understand how the main conclusions are significant or useful for other researchers in the field. The first conclusion "that lake- and marine-terminating glaciers flow, on average, 10% and 44% faster than land-terminating glaciers" (L21-22) is consistent with current understanding of glaciers and therefore not particularly noteworthy.

The second conclusion that "mass loss rates at lake-terminating glaciers are 57% greater than those at land- and marine-terminating glaciers" (L23-24) is potentially more noteworthy but not backed up with causal evidence that would lead to improved scientific understanding. I remain very skeptical that the difference in mass loss between lake- and land-terminating glacier categories is caused by enhanced ablation at ice-marginal lakes. It's entirely possible (if not probable) that the difference in mass loss is just a function of where these glaciers happen to be situated on Earth (something that Reviewer #3 also raised). This is important because the manuscript goes on to state the need "for improved representation of non-land glacier termini in models of glacier evolution and sea-level rise" (L24-25) which seems a bit premature given the manuscript provides no direct evidence that this mass loss is caused by ice-marginal lakes.

The “where on Earth” hypothesis is partially supported by Figure 3d which shows that regions with some of the highest specific mass loss rates do not contain marine-terminating glaciers (e.g. Central Europe, South East Asia, New Zealand). Likewise, shelf-terminating glaciers with the lowest specific mass loss rates are only found in two very cold places (e.g. Russian Arctic and Antarctic). This raises questions about whether the linear mixed-effects model has sufficiently controlled for climate. More lines of evidence would be required to dismiss this hypothesis such as side-to-side differences in mass loss between glaciers that are adjacent to each other but have different terminus types. Or a more direct analysis of ablation caused by ice-marginal lakes versus ice-atmosphere processes.

(Remarks on code availability)

Reviewer #3

(Remarks to the Author)
I appreciate the authors' careful responses to my previous comments and the substantial revisions made to the manuscript. In particular, I am pleased to see that the manuscript has been reframed to emphasize associations between terminus environment and glacier dynamics rather than implying direct causation. The revised title, more cautious language throughout, and expanded discussion of alternative explanations improves the interpretation of results.

Overall, I believe that the authors have adequately addressed my primary concerns. The manuscript now presents a valuable global synthesis of how glacier dynamics varies across terminus environments, while appropriately acknowledging the limitations of inferring mechanism from large-scale observational analyses. I support publication.

(Remarks on code availability)

Version 2:

Reviewer comments:

Reviewer #2

(Remarks to the Author)
I thank the authors for responding to my comments. I'm still not convinced that the linear mixed-effects model fully controls for geometry and climate given the massive differences in 1) sample sizes between land-terminating (n=209,563) vs. lake-terminating (3,542) and 2) areas of land-terminating (median=0.24 km2) vs. lake-terminating (median=2.00 km2) and 3) elevation of land-terminating (median-3,389 m) and lake-terminating (median=1,352 m) glaciers. I feel like the authors could have done more analysis to investigate this source uncertainty (e.g. interaction terms, loss of information in the PCs, extrapolation of linear relationships to steep, high-elevation regions where there are few lake-terminating glaciers), instead of just consistently claiming that their models "...control for climatic variability across all glaciers in the dataset...".

(Remarks on code availability)

Open Access This Peer Review File is licensed under a Creative Commons Attribution 4.0 International License, which permits use, sharing, adaptation, distribution and reproduction in any medium or format, as long as you give appropriate credit to the original author(s) and the source, provide a link to the Creative Commons license, and indicate if changes were made.
In cases where reviewers are anonymous, credit should be given to 'Anonymous Referee' and the source.
The images or other third party material in this Peer Review File are included in the article's Creative Commons license, unless indicated otherwise in a credit line to the material. If material is not included in the article's Creative Commons license and your intended use is not permitted by statutory regulation or exceeds the permitted use, you will need to obtain permission directly from the copyright holder.
To view a copy of this license, visit https://creativecommons.org/licenses/by/4.0/

Global-scale variability of glacier velocity and mass loss across terminus environments

Please note that line numbers in this response document correspond to those in the revised manuscript with track changes. For clarity, we have divided some longer reviewer comments using ellipsis, addressing each part in turn.

Reviewer #1 (Remarks to the Author):

Review of „Global-scale influence of terminus environment on glacier velocity and mass loss“ by Mallalieu et al.

The paper brings concise overview of the glacier terminus type and relate it to diverse glacier characteristics. It might be a useful dataset for further studies and a baseline for more developed studies.

The methods are clear and well described. I am just not sure why excluding surge type glacier when assessing glacier dynamics. Surging is part of the game and affects velocity and mass balance significantly. Perhaps it might be interesting to (at least) look at the surge type glaciers separately and provide an overview on distribution of surge type glaciers within the four categories. I guess the surge type glaciers will mostly fall in lake and certainly marine terminating glaciers category?...

RESPONSE: We thank the reviewer for this suggestion. The distribution of surge-type glaciers (following the Sevestre and Benn 2015 classification) is presented in Table R1 below **and now stated in the methods for transparency (line 366)**: *"…(comprising 0.6% of land-, 1.7% of lake-, and 3.8% of marine-termini respectively)"*.

Terminus environment	*Number of surge-type glaciers*	*% of all surge-type glaciers*	*% of all glaciers of that terminus type*
Land-terminating	1162	86.5	0.6
Lake-terminating	60	4.5	1.7
Marine-terminating	121	9.0	3.8
Shelf-terminating	0	0.0	0.0
All	1343	100	0.6

Table R1. Distribution of surge-type glaciers across terminus-types following the classification of Sevestre and Benn (2015).

To isolate the relationship between terminus environment and glacier dynamics we aimed to either control for, or remove, confounding variables from our statistical models. Accordingly, glaciers exhibiting dynamic behaviour potentially unrepresentative of local climate and topography were removed (including surging glaciers; lines 362-368). To assess the impact of this decision, we have re-run the statistical models (presented in Supplementary Tables 4-5) to include all surging glaciers, and present the outputs alongside the original model estimates in Tables R2-R3 of this response document. Across both the velocity (Table R2) and mass change rate (Table R3) models, estimates are consistent (typically to ~2 decimal places), indicating that the inclusion or exclusion of surging glaciers does not affect the overall results and interpretation presented in the manuscript. We therefore retain their exclusion because surging represents episodic, non-steady-state behaviour that is potentially unrepresentative of the terminus-type controls that are the focus of this study.

Independent variable	*Original model estimate**	*Model estimate including surging glaciers*
(Intercept)	0.612	0.613
Glacier terminus type: lake	0.042	0.041
Glacier terminus type: marine	0.158	0.155
Glacier morphometry PC	0.101	0.101
Median glacier elevation	-0.128	-0.129
Climate PC	0.027	0.026
n	175,925	177,232
R^2m	0.18	0.18
R^2c	0.38	0.38

Table R2. Comparison of linear mixed-effects model estimates for models including and excluding surging glaciers. Estimates show $\log_{10}$-transformed median glacier velocity as a function of terminus type and independent physical and climatic factors. *See supplementary Table 4.

Independent variable	*Original model estimate**	*Model estimate including surging glaciers*
(Intercept)	-0.294	-0.295
Glacier terminus type: lake	-0.162	-0.163
Glacier terminus type: marine	0.019	0.010
Glacier terminus type: shelf	0.100	0.087
Glacier elevation range	-0.018	-0.016
Median glacier elevation	0.033	0.033
Glacier area	-0.004	-0.003
Debris cover (%)	-0.012	-0.012
Polar deviation	0.014	0.014
Climate PC	-0.046	-0.047
n	196,747	198,023
R^2m	0.08	0.08
R^2c	0.28	0.28

Table R3. Comparison of linear mixed-effects model estimates for models including and excluding surging glaciers. Estimates show specific mass-change rate (2000-2019) as a function of terminus type and independent physical and climatic factors. *See supplementary Table 5.

…Also, can you comment on the highest velocity of the marine terminating glaciers in Alaska (Fig 3c) – isnt it affected by surging? Is it purely climatic driven?

RESPONSE: As noted above, Figure 3c excludes all surging glaciers, thus the relatively high median velocities of marine-terminating glaciers in Alaska are not the product of surging. Instead, these high velocities likely reflect local maritime and topographic conditions favourable for fast glacier flow, including high snowfall, relatively mild temperatures, and steeper surface slopes. We note that the Southern Andes RGI region, which shares similar characteristics, exhibits comparable marine-terminating glacier velocities (Fig. 3c).

Another issue worth exploring (at least discussing) might be to turn the problem around – for example the large and fast flowing glaciers often reach the marine environment. So the terminus type (marine in this case) might also be a result of the high glacier dynamics, not only the cause of the high dynamics. On the contrary, small glaciers usually exhibit low dynamics and do not reach marine environment or develop ice-contact lakes. I think you mentioned similar issue while talking about the lake-terminating type and the effect of topography. Can you comment on that?

RESPONSE: We thank the reviewer for this suggestion and agree that terminus type could in some cases reflect underlying glacier dynamics. **We have added a sentence to acknowledge this point (lines 213-215):** *"It is also possible that terminus type partly reflects underlying glacier dynamics (with larger, faster-flowing systems more likely to reach marine settings)."*

I think the paper reads well, I dont have any detailed comments to the text itself. Also I cannot comment on the language as unlike most of the authors I am not a native English language speaker.

Thanks for taking my comments/suggestions into consideration, I hope it might help!

We thank the reviewer for their constructive feedback and suggestions.

Reviewer #2 (Remarks to the Author):

The goal of this manuscript is to quantify the influence of terminus type of dynamics of mountain glaciers, namely velocity and mass change. To do this, the authors categorize mountain glaciers defined by the Randolph Glacier Inventory (RGI) version 6.0 as land-, lake-, or marine-terminating. The main findings are that lake- and marine-terminating glaciers flow faster than land-terminating glaciers (not that surprising) and that lake-terminating glaciers have higher specific mass loss rates than both land- and marine-terminating glaciers (more interesting). The overall quality of the presentation is good. The manuscript is well-written and the figures and tables clear communicate the findings. That said, I do have two major concerns. The first is about the methods that the authors use to assess the impact of lakes on glaciers. The second is that approach used to separate glaciers into lake-terminating category. In my opinion these issues seriously undermine the robustness of the findings and for this reason I recommend that the manuscript is rejected.

Major comments

My first concern is that the current statistical analysis does not sufficiently support the primary goal of the study which is "to quantify… the influence of terminus type on glacier dynamics at the global scale" (L51-52). Since the authors do not directly measure the mass loss caused by ice-marginal lakes, the analysis cannot differentiate between mass loss caused by ice-marginal lakes from that caused by ice-atmosphere interactions. I think the authors are well aware of this limitation. But the problem is that the statement in the abstract that reads "…mass loss rates at lake-terminating glaciers are 57% greater than those at land- and marine-terminating glaciers" is deceptive because the analysis provides no evidence that lakes are actually responsible for these differences (even though it is strongly implied). Without a more direct attribution of glacier mass loss to processes occurring at the terminus, the conclusions of the manuscript are relatively weak e.g. "non-land termini have the potential to exert a disproportionate influence on glacier dynamics" (L152-153) and "glacier terminus types are associated with distinct velocity and specific mass change regimes" (L214-215). Unfortunately these statements are lacking the causality required to fulfil the ambitious goal of the study and contribute to scientific understanding.

RESPONSE: We agree that, as an observational study, our analysis quantifies associations between terminus type and glacier dynamics rather than demonstrating direct causation. **We have therefore moderated causal language throughout the revised manuscript accordingly, including: replacing 'influence' with 'relationship' or 'association' where referring to our own objectives and results; removing direct causal attribution language from the opening and closing paragraphs of the Discussion; revising the captions of Supplementary Tables 4**

and 5; and revising the title to "Global-scale variability of glacier velocity and mass loss across terminus environments".

We note, however, that direct measurement of terminus-specific processes such as calving flux and subaqueous melt rates at the global scale is not currently feasible and is not a prerequisite for quantifying statistical associations between terminus type and glacier dynamics. Our linear mixed-effects modelling framework represents a robust inferential approach for a global-scale study of this nature. Specifically, by controlling for key physical and climatic drivers of glacier dynamics (including temperature, precipitation and glacier morphology), our statistical models estimate the independent associations between terminus type and glacier velocity and mass change, thereby accounting for key confounding environmental factors. In particular, by including climate as a covariate, our models account for the primary source of ice-atmosphere driven mass loss, allowing the independent association between terminus type and mass change to be estimated separately from climatic influences. We are furthermore confident that our results reflect genuine physical relationships rather than statistical artefacts, because our global-scale findings are consistent with well-documented mechanisms by which lacustrine and marine environments influence glacier dynamics, including reduced back-pressure from calving, subaqueous melting, and thermo-mechanical lake-glacier interactions (as detailed in paragraphs 2-3 of the Discussion). This consistency between our statistical associations and independently documented physical mechanisms provides additional confidence that terminus type is independently associated with glacier dynamics.

The abstract statement highlighted by the reviewer (lines 23-24) reports an observed difference in mass loss rates between terminus types after accounting for key climatic and geographic influences; it does not make a direct causal attribution to lakes. We therefore consider this statement to be an appropriate and accurate summary of our results. Quantifying and reporting such associations is the central objective of this study and is consistent with standard practice in regional- to global-scale observational approaches in glaciology, which routinely use statistical analyses to infer physically meaningful controls without directly measuring all underlying processes (e.g. Dehecq et al. 2019; Sevestre and Benn 2015; Mallalieu et al. 2021).

We also note that the two conclusions highlighted by the reviewer (lines 160-162 and 233-235, respectively) are deliberately framed in cautious, associative language (e.g., *"have the potential to"* and *"are associated with"*) that reflects the strength of evidence provided by our analysis and is consistent with the language moderation outlined above. Strengthening these statements to make direct causal claims would not be supported by our observational analysis and would therefore be inconsistent with the reviewer's concern that the manuscript should avoid implying causation without sufficient evidence. We therefore consider these conclusions to accurately reflect the level of inference supported by our analysis. To our knowledge, this study provides the first global-scale quantification of associations between terminus type and glacier dynamics. These associations provide an empirical foundation for guiding future process-based investigations and improving the representation of terminus-specific dynamics in models of glacier evolution and sea-level rise.

My second major concern is that the classification scheme likely overestimates the number of glaciers that are actually impacted by ice-marginal lakes. The study classifies lake-terminating glaciers as those in the RGI inventory which are within 60 m of a lake identified by the global glacier lake inventory developed by Shugar et al. and no more than 100 m above the minimum glacier elevation. However, following this approach, a glacier with a small lake near the side of its terminus would be classified as lake-terminating, even if the lake has very little influence on its dynamics. Inclusion of this "lake-terminating" (but not really) glacier into the subsequent statistical analysis, is likely to skew the velocity and mass change of the lake-terminating category. Currently the authors provide no discussion of how this scenario would impact their findings. A better approach would be to determine the influence of ice-marginal lakes more directly. This is what the Steiner et al. (2025) paper does for

example by looking for evidence of calving into ice-marginal lakes. I realize that modifying the analysis would be challenging at this stage but this is a major flaw of the current approach.
RESPONSE: We note that Steiner et al. (2026) classify lake-terminating glaciers for RGI 7.0 using a different methodology, further dividing their lake-termini into three sub-categories intended to reflect the degree of lake impact on glacier dynamics. However, these sub-categories are defined by geometric criteria requiring contributor judgement, specifically percentage thresholds of terminus contact, with visual indicators of dynamic activity such as calving serving only as secondary observations. Allocation of glaciers into these sub-categories also relies on visual assessments and further contributor judgement - the subjective nature of which is acknowledged in Steiner et al. (2026) - and there is no quantitative evidence that glaciers in the different sub-categories exhibit dynamically distinct behaviour.

Our manuscript instead aims to quantify associations between glacier dynamics and first-order terminus categories (land, lake, marine, shelf) using a fully replicable and objective approach. Rather than making judgements about the degree to which individual lakes influence glacier dynamics, we classify all glaciers with terminus lakes as lake-terminating - an approach that is internally consistent and does not require assumptions about lake impact that are currently unsupported. We also note that the spatial and elevational filters applied in our classification are specifically designed to exclude lakes not in meaningful contact with a glacier terminus. Furthermore, our use of the Shugar et al. (2020) global glacier lake inventory imposes a minimum lake size threshold (≥0.05 km^2) that is five times more conservative than that used by Steiner et al. (2026) (≥0.01 km^2), meaning that very small lakes do not result in glaciers acquiring lake-terminating status in our classification. **We have added text to the Discussion to address the potential impact of this approach on our reported results (lines 171-174):** *"We also note that our lake-terminating category intentionally includes all glaciers with terminus lakes, regardless of the degree of lake contact. Glaciers with limited lake interaction may therefore exhibit dynamics closer to land-terminating glaciers, suggesting our reported differences in dynamics between lake- and land-terminating glaciers are likely conservative estimates."*

We agree that quantifying the extent to which individual glacier dynamics are impacted by their respective lakes is a valuable line of enquiry arising from both studies. In addition to the lake area and lake - ice contact variables highlighted by Steiner et al. (2026), such work would also need to consider the role of additional lake variables, including depth, temperature, persistence, and growth/contraction. However, such analyses lie beyond the existing scope of our manuscript, which concerns differences in inter- rather than intra-terminus type glacier dynamics. **We have added the following text to the Discussion to acknowledge this as an important direction for future research (lines 201-203):** *"Quantifying the influence of individual lakes on glacier dynamics - and the role of associated lake properties including area, depth, temperature, persistence and lake-ice interface length - represents an important direction for future research."*

The authors justify the number of glaciers impacted by ice-marginal lakes by providing a coarse comparison between the percentage of lake-terminating glaciers identified by their approach relative to Steiner et al. (2025). However, they do not mention that Steiner et al. (2025) differentiates lake-terminating glaciers into three classes, the third of which contains glaciers that "do not have visible impacts due to the lake except along a very limited (<10%) portion of the terminus"…
RESPONSE: We thank the reviewer for this comment, which prompted a more explicit and transparent comparison of our global lake-terminating glacier statistics with those of Steiner et al. (2026), presented in Table R4 below. Our values are more conservative than those reported for categories 1, 2 and 3 combined (i.e. all glaciers determined by Steiner et al. 2026 to have a lake at their terminus), likely arising from the more restrictive minimum lake size threshold used in our classification (≥0.05 km^2 versus ≥0.01 km^2 in Steiner et al. 2026). However, our values are in good agreement with those reported for categories 2 and 3 only (i.e. those glaciers considered by Steiner et al. 2026 likely to be dynamically impacted by their terminus lakes, and thus retained as lake-terminating in RGI 7.0), suggesting our more restrictive lake size threshold effectively filters out smaller

lakes that would be unlikely to meet the >10% contact threshold for retention in the final lake-termini classification presented in Steiner et al. (2026). The close correspondence between our values and those of Steiner et al. (2026) categories 2-3 therefore suggests our classification does not substantially overestimate the global extent of lake-terminating glaciers likely to be dynamically impacted by their terminus lakes. **To improve the clarity and transparency of our comparisons, Table R4 has been added to the manuscript (as Table 2) and the associated text updated to acknowledge that Steiner et al. (2026) present sub-classes of lake-terminating glaciers, and specify the exact sources of our comparative values (lines 339-345).**

	This study: RGI 6.0 lake-terminating glaciers	*Steiner et al. (2026): RGI 7.0 lake-terminating glaciers*	
		Categories 2 and 3	*Categories 1, 2 and 3*
Global glaciers terminating in a lake (%)	1.6	1.4	1.9
Global glacier area terminating in a lake (%)	11.5	11.4	15.7
Global glacier area terminating in a lake (10^3 km^2)	86.0	80.8	111.4

Table R4. Comparison of global lake-terminating glacier statistics between this study and Steiner et al. (2026). Values for Steiner et al. (2026) represent the sum of the indicated categories.

We also note an inconsistency in the original manuscript, in which our global values were compared to Steiner et al. (2026) categories 2 and 3 combined, whereas regional values for Alaska and the Southern Andes were compared to categories 1, 2 and 3 combined. For consistency, we have updated the comparison of our regional values to Steiner et al. (2026) categories 2 and 3 only (Alaska 0.7%; Southern Andes 1.9%; lines 353). Our slightly higher regional values (0.9% and 2.3% respectively) likely reflect the longer time frame over which glaciers could acquire lake-terminating status in our classification, as noted in the manuscript (lines 345-349).

…For the same reason, it is perhaps not surprising that the authors identify 68% and 174% more lake-terminating glaciers in Alaska and Southern Andes (L323-324) than RGI 6.0…
RESPONSE: We note that the percentage values of lake-termini in Alaska and the Southern Andes reported in our study and by Steiner et al. (2026) are comparable (lines 351-354). Our higher values relative to RGI 6.0 in Alaska and the Southern Andes reflect the incompleteness of the original RGI 6.0 lake-terminus classification (*TermType*) within the regions in which lake-terminating glaciers were identified, rather than an overestimate of lake-termini in our classification (as noted in lines 354-355). Equivalent calculations from the reported data in Steiner et al. (2026) also indicate similarly high values of lake-termini in these regions relative to the original RGI 6.0 terminus classification. **We have made minor amendments to the manuscript text to improve the clarity of this comparison (lines 349-351).**

…The problem is that the narrative of the manuscript implies that ice-marginal lakes cause glaciers to flow 10% faster and lose 57% more mass. But these values are unlikely to be accurate if the numbers (and parts) of glaciers that are impacted by lakes are dramatically overestimated.
RESPONSE: As detailed in our response to the earlier comment, causal language has been moderated throughout the revised manuscript, with reported differences in dynamics across terminus types now framed explicitly as statistical associations rather than causal relationships. On the accuracy of our reported associations, we note that any glaciers with limited lake contact at their terminus are likely to exhibit dynamics closer to those of land-terminating glaciers, thereby moderating rather than accentuating the observed differences between terminus types - meaning our reported associations are if anything conservative estimates. Furthermore, even where borderline cases exist, our large-sample linear mixed-effects modelling framework is designed to detect systematic associations across the full dataset - any influence of borderline cases on the lake-terminating category would pull its dynamic properties towards those of land-terminating glaciers, making the true differences between terminus types harder to detect rather than easier, and providing further confidence that our reported associations are robust.

Specific comments

L39: It would be useful to clarify somewhere in this paragraph that these glacier evolution models are for "mountain" glaciers as opposed to tidewater glaciers flowing from ice sheets which do account for ice dynamics.
RESPONSE: *'Mountain'* **has been added to line 41** to make this distinction clear.

L43-44: Missing from this paragraph is a couple of statements that review the known differences between dynamics of land- vs lake- vs marine-terminating glaciers.
RESPONSE: We thank the reviewer for this suggestion and **have added text (lines 45-47)** drawing on regional observational studies to acknowledge known dynamical differences between terminus types: *"Regional observations indicate that lake- and marine-terminating glaciers can exhibit distinct dynamic behaviours relative to their land-terminating counterparts, including faster ice flow and enhanced dynamic thinning*[17,26-31]*, but the global-scale expression of these differences remains unquantified."*

L108-110: I don't think that these statistics are very useful since they are not normalized for area. The fact that bigger, marine-terminating glaciers flow faster than smaller land- and lake-terminating glaciers is self-evident.
RESPONSE: Glacier velocity is a rate (m a^{-1}) and not an area-integrated quantity, so normalisation by glacier area is not typically applied. In contrast, quantities such as mass loss depend on glacier size and are therefore expressed in area-normalised form (i.e. specific mass balance). We agree that glacier size and geometry can influence flow speed, and that larger marine-terminating glaciers often exhibit higher velocities. Later in the Results, we account for these influences on velocity by using a linear mixed-effects model that includes glacier morphometry as a control variable (represented by a principal component of glacier slope and thickness, both of which correlate strongly with glacier area) and present the modelled results in Fig. 4. However, we retain these raw statistics as they represent, to our knowledge, the first global estimates of median glacier velocity by terminus type, providing a novel descriptive baseline that complements the controlled statistical analysis presented in Fig. 4a and Supplementary Table 4.

L135-136: It's not clear why the volume or area of the glacier is also not accounted for.

RESPONSE: Glacier velocity is primarily controlled by ice thickness, surface slope and climatic conditions, which are captured in our model through the morphometry and climate covariates. Glacier area and volume were not included as predictors because: i) they are not direct physical controls on glacier flow within our modelling framework, and ii) they are strongly correlated with variables already included in the model (particularly ice thickness, represented in the morphometry principal component). To assess sensitivity to this choice, we have tested an alternative model that includes glacier area as a covariate (Table R5), which produces near-identical estimates and measures of model fit (to at least two decimal places), and thus does not affect our results or their interpretation. We therefore retain the more parsimonious model formulation in the main manuscript.

Independent variable	*Original model estimate**	*Model estimate including area*
(Intercept)	0.612	0.612
Glacier terminus type: lake	0.042	0.040
Glacier terminus type: marine	0.158	0.152
Glacier morphometry PC	0.101	0.100
Median glacier elevation	-0.128	-0.128
Climate PC	0.027	0.027
**Glacier area**	NA	0.003
n	175,925	175,925
R^2m	0.18	0.18
R^2c	0.38	0.38

Table R5. Comparison of linear mixed-effects model estimates for models including and excluding area as a covariate. Estimates show log_{10}-transformed median glacier velocity as a function of terminus type and independent physical and climatic factors. *See supplementary Table 4.

L378-379: I don't think this conforms to the reporting standards for Nature Portfolio journals which require code and data to be available to reviewers during peer review process

RESPONSE: We confirm that both the code and data were made available during the peer review process via the manuscript tracking system, in accordance with the journal's reporting standards (as a Readme file and Datasets 1 and 2, respectively). We are sorry that access issues were encountered and will ensure that access is clarified further in the revised submission. We remain happy to assist if any problems persist.

References

Steiner et al. (2025) https://essd.copernicus.org/preprints/essd-2025-315/

Reviewer #2 (Remarks on code availability):

The authors did not make the code (or data) available to the reviewers.

RESPONSE: We confirm again that both the code and data were made available to reviewers via the manuscript tracking system during peer review, in accordance with journal requirements.

Reviewer #3 (Remarks to the Author):

This manuscript provides a valuable and timely update to prior global synthesis studies of glacier mass and volume change estimates by examining how terminal environment plays a role in explaining variation in specific mass change. Their main findings are that marine-terminating glaciers are faster than their land-terminating counterparts, but they lose less mass than land-terminating glaciers. They also find that lake-terminating glaciers

lose the most mass – even when examined regionally (for most regions). The authors claim that the reasons for this increase in mass loss for lake-terminating glaciers results from enhanced terminus-lake interaction and the unique terminus geometry of lake-terminating glaciers that permits longer response times.

I am, however, not convinced that the results presented here are at the level of Nature Communications for a few reasons. First, the authors do not demonstrate cause and effect. They provide compelling correlations, but the physical mechanisms remain inferred, not demonstrated…

RESPONSE: We agree that, as an observational study, our analysis quantifies associations between terminus type and glacier dynamics rather than demonstrating direct causation. **We have therefore moderated causal language throughout the revised manuscript accordingly, including: replacing 'influence' with 'relationship' or 'association' where referring to our own objectives and results; removing direct causal attribution language from the opening and closing paragraphs of the Discussion; revising the captions of Supplementary Tables 4 and 5; and revising the title to *"Global-scale variability of glacier velocity and mass loss across terminus environments".***

We note, however, that our linear mixed-effects modelling framework represents a robust inferential approach for a global-scale study of this nature. Specifically, by controlling for key physical and climatic drivers of glacier dynamics (including temperature, precipitation and glacier morphology), our statistical models estimate the independent associations between terminus type and glacier velocity and mass change, thereby accounting for key confounding environmental factors. We are furthermore confident that our results reflect genuine physical relationships rather than statistical artefacts, because our global-scale findings are consistent with well-documented mechanisms by which lacustrine and marine environments influence glacier dynamics, including reduced back-pressure from calving, subaqueous melting, and thermo-mechanical lake-glacier interactions (as detailed in paragraphs 2-3 of the Discussion). This consistency between our statistical associations and independently documented physical mechanisms provides additional confidence that terminus type is independently associated with glacier dynamics.

…Further, Table 1 shows that lake-terminating glaciers occupy warmer environments than other terminus types. Is this intrinsic to this terminus environment or a consequence of other geographic factors, like elevation and latitude, both of which strongly influence environmental temperature. The current manuscript makes it difficult to assess if the reported differences in mass loss by terminus type are simply a consequence of where these different terminus types are found.

RESPONSE: To address the specific concern regarding lake-terminating glaciers occupying warmer environments, we note that the mass change statistical model explicitly includes a climate principal component - capturing covariance in mean annual air temperature and total annual precipitation - alongside median glacier elevation and other key influences on mass change (Supplementary Table 5). The results presented in lines 143-150 and Fig. 4 of the manuscript are therefore robust to the climatic and geographic differences between terminus types, and the warmer environments occupied by lake-terminating glaciers on average (Table 1) do not explain the differences in mass change (or velocities) observed between terminus types. **We have, however, revised lines 141-143 of the manuscript** to explicitly stress that the reported terminus-type effects are estimated after accounting for climatic, geometric and topographic differences between terminus types: *"Linear mixed-effects statistical models were applied to isolate associations between terminus type and glacier dynamics whilst controlling for other key climatic, geometric and topographic influences (see Methods)."*

In addition, because lakes are smaller volumes, and these glaciers are generally in warmer conditions than marine-terminating glaciers I would expect that the water temperature near the terminus of lake-terminating glaciers to be warmer than marine-terminating counterparts. Lakes, unlike fjords, can more rapidly warm and store seasonal heat and this may explain why they lose more mass. The manuscript does not adequately discuss this point.

RESPONSE: We thank the reviewer for this suggestion. We agree that the thermal response of ice-marginal lakes, including their relatively small volume and potential for seasonal heat storage, may contribute to enhanced frontal ablation. **We have added a sentence to acknowledge this mechanism (lines 196-198):** *"In addition, proglacial lakes may respond rapidly to atmospheric forcing due to their relatively small volume and limited water exchange, potentially allowing enhanced seasonal heat storage at the glacier terminus, which could further promote frontal ablation[46-48]."*

Some smaller points –

• I'm not sure what a shelf-terminating glacier is – perhaps because these are exclusively in the Antarctic, these are glaciers that terminate in an ice shelf? I'm not sure they add much value to the manuscript – they are not discussed at length and their population is small and regional.

RESPONSE: Shelf-terminating glaciers, defined as glaciers that terminate in an ice shelf following the RGI 6.0 classification (RGI Consortium 2017), are limited to the Antarctic and Russian Arctic RGI regions. Whilst these glaciers comprise a relatively small component of our dataset, we retain them to meet our objective of providing a globally-complete classification and analysis of glacier termini, including less common terminus types.

• 154: "they collectively account for more than half of global glacier area and mass loss (Fig 1)" – but Figure 1 does not show mass loss, just area and there is no global accounting, just regional. It would be good to provide the data on this statement, since it constitutes most of their thesis. What I'm asking for is lake-terminating glacier area/global glacier area and lake-terminating glacier mass loss/global glacier mass loss. You could make a global area pie chart for Fig. 1 for the first request and then do some calculations to provide the stats for the latter. This would allow us to see how much non-land-terminating glaciers drive the global average mass loss estimates.

RESPONSE: We thank the reviewer for this suggestion. Figure 1 presents the global distributions of glacier number, glacier area, and mass change (negative values corresponding to mass loss) by terminus type, shown as both absolute and relative (percentage) contributions, whereas regional distributions of glacier area by terminus type are shown separately (Fig. 2). The statement in lines 160-162 refers directly to these global values presented in Fig. 1, which show that non-land termini collectively account for 53.8% of global glacier area and 50.6% of global mass change (2000-2019 rate). We retain the current figure format, which pairs absolute values with percentage bars, as this conveys both the absolute magnitude and relative contribution of each terminus type across all three variables.

• 189: reduced gradients in what?

RESPONSE: Gradient in ice surface slope. **The text on line 206 has been amended for clarity:** *"…and with reduced ice surface slopes in their lowermost regions…"*

REFERENCES

Dehecq, A., Gourmelen, N., Gardner, A.S. *et al.* Twenty-first century glacier slowdown driven by mass loss in High Mountain Asia. *Nature Geosci* **12**, 22-27 (2019).

Mallalieu, J., Carrivick, J.L., Quincey, D.J. and Raby, C.L. Ice-marginal lakes associated with enhanced recession of the Greenland Ice Sheet. *Global and Planetary Change* **202**, 103503 (2021).

RGI Consortium. Randolph Glacier Inventory (RGI) - A Dataset of Global Glacier Outlines: Version 6.0. NSIDC: National Snow and Ice Data Center. https://doi.org/10.7265/4m1f-gd79 (2017).

Sevestre, H. & Benn, D. I. Climatic and geometric controls on the global distribution of surge-type glaciers: implications for a unifying model of surging. *Journal of Glaciology* **61**, 646–662 (2015).

Shugar, D. H. et al. Rapid worldwide growth of glacial lakes since 1990. Nat. Clim. Chang. 10, 939–945 (2020).

Steiner, J. et al. Global mapping of lake-terminating glaciers. Earth System Science Data 18, 1665–1681 (2026).

Global-scale variability of glacier velocity and mass loss across terminus environments

Reviewer #1 (Remarks to the Author):

dear authors,

thanks for clarifying the issues raised in my previous review, I was expecting the surging to be more important. I think the article reads well now and the additions made on request of other reviewers helped as well. I guess, the manuscript is ready for publication now.

with regards,
Jan

RESPONSE: We thank the reviewer for their constructive comments in the earlier round of revision.

Reviewer #2 (Remarks to the Author):

I thank the authors for responding to my comments. The updated manuscript has been lightly revised. The main changes are that much of the casual language between terminus type and mass loss has been moderated. The manuscript also provides a more direct comparison with a previous study which quantified the terminus types of global glaciers e.g. Table 2 (Steiner et al., 2026).

RESPONSE: We note that, in addition to moderating causal language throughout the manuscript, the stated aim of the study was revised in round 1 from quantifying 'the influence of terminus type on glacier dynamics' to quantifying 'the associations between terminus type and glacier dynamics'. This distinction is central to interpreting the revised manuscript's conclusions, several of which appear to be assessed against the original rather than revised aim in this round of review. We also note that Steiner et al. (2026) presented a classification of lake-terminating glaciers only, not all terminus types globally - the latter remaining one of the primary novel contributions of our study.

Despite the slight reframing of the manuscript, I'm still struggling to understand how the main conclusions are significant or useful for other researchers in the field. The first conclusion "that lake- and marine-terminating glaciers flow, on average, 10% and 44% faster than land-terminating glaciers" (L21-22) is consistent with current understanding of glaciers and therefore not particularly noteworthy.

RESPONSE: This comment was raised and addressed in round 1 of revisions. We retain these values as they represent, to our knowledge, the first global estimates of median glacier velocity by terminus type. More broadly, the study's contributions - including the first globally-complete classification of glacier termini, the first global quantification of specific mass change rates by terminus type, and the finding that non-land termini account for more than half of global glacier area and mass loss - provide an empirical foundation for global glacier evolution modelling and a first-order estimate of the potential dynamic consequences of ongoing terminus transitions (lines 215-218 and 233-236).

The second conclusion that "mass loss rates at lake-terminating glaciers are 57% greater than those at land- and marine-terminating glaciers" (L23-24) is potentially more noteworthy but not backed up with causal evidence that would lead to improved scientific understanding. I remain very skeptical that the difference in mass loss between lake- and land-terminating glacier categories is caused by enhanced ablation at ice-marginal lakes. It's entirely possible (if not probable) that the difference in mass loss is just a function of where these glaciers happen to be situated on Earth (something that Reviewer #3 also raised). This is important because the manuscript goes on to state the need "for improved representation of non-land glacier termini in models of glacier evolution and sea-level rise" (L24-25) which seems a bit premature given the manuscript provides no direct evidence that this mass loss is caused by ice-marginal lakes.

RESPONSE: These concerns were addressed in round 1 of revisions. The revised manuscript quantifies associations between terminus type and glacier dynamics - it does not aim to demonstrate direct causation, nor do the results, conclusions or abstract make direct causal attributions of differences in glacier dynamics to terminus-specific processes. Quantifying and reporting associations without demonstrating direct causation is consistent with standard practice in regional- to global-scale observational glaciology, as detailed with supporting citations in our round 1 response. We note that causal language was moderated throughout the manuscript in response to reviewer feedback in round 1, and that the revised manuscript's conclusions are framed accordingly.

The concern that mass loss differences reflect geographic distribution rather than terminus-type associations was raised by Reviewer #3 in round 1 and addressed in detail in our response. We note Reviewer #3 is now satisfied and supports publication. As outlined in our original response, the mass change statistical model explicitly includes a climate principal component that captures covariance in mean annual air temperature and total annual precipitation for every glacier in the dataset (n = 196,747), alongside other key geometric and topographic influences on glacier dynamics (Supplementary Table 5). Our results are therefore robust to the climatic and geographic differences between terminus types, and show that terminus types are associated with distinct dynamic regimes regardless of the underlying mechanism - the recommendation for improved model representation follows directly from these findings.

The “where on Earth” hypothesis is partially supported by Figure 3d which shows that regions with some of the highest specific mass loss rates do not contain marine-terminating glaciers (e.g. Central Europe, South East Asia, New Zealand). Likewise, shelf-terminating glaciers with the lowest specific mass loss rates are only found in two very cold places (e.g. Russian Arctic and Antarctic). This raises questions about whether the linear mixed-effects model has sufficiently controlled for climate. More lines of evidence would be required to dismiss this hypothesis such as side-to-side differences in mass loss between glaciers that are adjacent to each other but have different terminus types. Or a more direct analysis of ablation caused by ice-marginal lakes versus ice-atmosphere processes.

RESPONSE: We agree that the distribution of some terminus types (particularly marine- and shelf-termini) reflects climatic setting; however, as noted above, our statistical models control for climatic variability across all glaciers in the dataset. We also note that intra-regional comparisons are more meaningful than inter-regional comparisons, with Figure 3d showing that lake-termini exhibit more negative specific mass change rates than other terminus types in 15 of the 18 RGI regions with available data (lines 119-121), thus directly contradicting the 'where on Earth' hypothesis. Furthermore, Fig. 4b and Supplementary Table 5 already present the suggested side-to-side glacier comparison, but at a global scale - showing the associations between terminus type and mass change rate whilst holding climate, morphometry and other key influences on glacier dynamics constant across 196,747 glaciers (~91% of those in RGI 6.0). Paired comparisons of adjacent glaciers would be both less rigorous and less representative. A direct analysis of ablation caused by ice-marginal lakes versus ice-atmosphere processes at the global scale is not feasible due to limited field data and is beyond the scope of this study, which explicitly aims to quantify associations rather than demonstrate causation.

Reviewer #3 (Remarks to the Author):

I appreciate the authors' careful responses to my previous comments and the substantial revisions made to the manuscript. In particular, I am pleased to see that the manuscript has been reframed to emphasize associations between terminus environment and glacier dynamics rather than implying direct causation. The revised title, more cautious language throughout, and expanded discussion of alternative explanations improves the interpretation of results.

Overall, I believe that the authors have adequately addressed my primary concerns. The manuscript now presents a valuable global synthesis of how glacier dynamics varies across terminus environments, while

appropriately acknowledging the limitations of inferring mechanism from large-scale observational analyses. I support publication.

RESPONSE: We thank the reviewer for their constructive comments in the earlier round of revision and their recognition of the contribution and value of our study.

Global-scale variability of glacier velocity and mass loss across terminus environments

Reviewer #2 (Remarks to the Author):

I thank the authors for responding to my comments. I'm still not convinced that the linear mixed-effects model fully controls for geometry and climate given the massive differences in 1) sample sizes between land-terminating (n=209,563) vs. lake-terminating (3,542) and 2) areas of land-terminating (median=0.24 km2) vs. lake-terminating (median=2.00 km2) and 3) elevation of land-terminating (median-3,389 m) and lake-terminating (median=1,352 m) glaciers. I feel like the authors could have done more analysis to investigate this source uncertainty (e.g. interaction terms, loss of information in the PCs, extrapolation of linear relationships to steep, high-elevation regions where there are few lake-terminating glaciers), instead of just consistently claiming that their models "...control for climatic variability across all glaciers in the dataset...".

RESPONSE: We thank the reviewer for this comment. The sample imbalance between terminus types reflects global glacier distribution rather than any analytical bias; we note that lake-terminus type coefficients remain highly significant ($p<0.001$) with narrow confidence intervals across both the velocity and mass change models (Supplementary Tables 4-5), demonstrating that the smaller lake-terminating sample does not undermine the reliability of our results.

The highlighted geometric differences between terminus types are explicitly addressed in our model design: the mass change model includes glacier area and median glacier elevation as covariates, while the velocity model similarly includes median glacier elevation as a covariate, and the sensitivity analysis presented in our round 1 response (Table R5) demonstrated that the inclusion of glacier area in the velocity model made no meaningful difference to the model outputs. We note that controlling for these geometric differences does not require the terminus type groups to be similar in area or elevation; rather, the models estimate the terminus type effect by holding these variables constant - effectively comparing glaciers of equivalent size and elevation across terminus types, regardless of the actual distributional differences between groups.

Regarding the suggested additional analyses: interaction terms were not included as we had no theoretical basis for expecting the relationships between glacier dynamics and model covariates to differ systematically across terminus types, and including them without such justification would add model complexity without improving interpretability; the morphometry and climate PCs retain 74% and 92% of the variance in their respective input variables (as noted in the Methods), representing minimal information loss; and lake-terminating glaciers occur across a wide range of elevations globally - for example, filtering the available dataset reveals that of the 3,384 lake-terminating glaciers in the mass change model, 484 of them are located in High Mountain Asia (RGI regions 13-15) with median elevations at or above 5,000 m a.s.l.
